# Polymer Electrolytes for Lithium-Ion Batteries Studied by NMR Techniques

**DOI:** 10.3390/membranes12040416

**Published:** 2022-04-11

**Authors:** Vitaly I. Volkov, Olga V. Yarmolenko, Alexander V. Chernyak, Nikita A. Slesarenko, Irina A. Avilova, Guzaliya R. Baymuratova, Alena V. Yudina

**Affiliations:** 1 Institute of Problems of Chemical Physics RAS, 142432 Chernogolovka, Russia; oyarm@mail.ru (O.V.Y.); sasha_cherniak@mail.ru (A.V.C.); wownik007@mail.ru (N.A.S.); irkaavka@gmail.com (I.A.A.); guzalia.rb@yandex.ru (G.R.B.); gvinok@yandex.ru (A.V.Y.); 2 Scientific Center in Chernogolovka RAS, 142432 Chernogolovka, Russia

**Keywords:** lithium-ion batteries, nanocomposite polymer electrolytes, ionic conductivity, electrochemical impedance, NMR spectroscopy, pulsed-field gradient NMR

## Abstract

This review is devoted to different types of novel polymer electrolytes for lithium power sources developed during the last decade. In the first part, the compositions and conductivity of various polymer electrolytes are considered. The second part contains NMR applications to the ion transport mechanism. Polymer electrolytes prevail over liquid electrolytes because of their exploitation safety and wider working temperature ranges. The gel electrolytes are mainly attractive. The systems based on polyethylene oxide, poly(vinylidene fluoride-co-hexafluoropropylene), poly(ethylene glycol) diacrylate, etc., modified by nanoparticle (TiO_2_, SiO_2_, etc.) additives and ionic liquids are considered in detail. NMR techniques such as high-resolution NMR, solid-state NMR, magic angle spinning (MAS) NMR, NMR relaxation, and pulsed-field gradient NMR applications are discussed. ^1^H, ^7^Li, and ^19^F NMR methods applied to polymer electrolytes are considered. Primary attention is given to the revelation of the ion transport mechanism. A nanochannel structure, compositions of ion complexes, and mobilities of cations and anions studied by NMR, quantum-chemical, and ionic conductivity methods are discussed.

## 1. Introduction

Today, there is a great demand for highly efficient mobile energy storage devices. They must permanently be improved from economic and environmental points of view and must be safe.

It should be noted that currently existing lithium-ion batteries (LIB) have problems ensuring the safety of their operation and high cost. Attempts to solve these problems have led to the emergence of lithium-ion batteries with a polymer electrolyte, which contains nonvolatile components and does not react with electrode materials [1,2]. In addition, this work is aimed at developing post-lithium systems based on sodium [3,4]. However, they would not solve all LIB problems, especially safety-related ones [5].

The use of polymer electrolytes (PE) in LIB radically solves the safety problem because the use of liquid electrolytes can be associated with self-ignition and even explosion. Solid PEs of the polymer–salt composition in most cases have no acceptable conductivity of 10^−3^ S/cm at room temperature and, therefore, at present, there are especially frequent studies of polymer gel electrolytes, as well as nanocomposites based on them.

Polymer gel electrolytes contain aprotic solvents, which can also be unsafe. As an alternative to volatile solvents, ionic liquids have recently begun to be used and, being molten salts, do not ignite and have no saturated vapor pressure. The presence of ionic liquids (IL) in the electrolyte significantly increases the operating temperature range. Therefore, the use of ILs in the composition of both liquid and polymer electrolytes is very important [6,7,8].

Another promising class of electrolytes for safe solid-state electrochemical devices is nanocomposite polymer electrolytes (NPEs). Such materials have a wide operating temperature range, provide high ionic conductivity and good physical and mechanical properties, and can serve as separators. Some types of NPE can be obtained by casting from a solution of polymer–lithium salt–nanopowder (SiO_2_, TiO_2_, etc.) followed by the removal of the inert solvent. The resulting dry membrane is then soaked in a liquid electrolyte. There is another class: nanocomposite network polymer gel electrolytes obtained by the reaction of radical polymerization directly in a liquid electrolyte [9,10,11,12].

All electrolytes that operate at ambient temperature can be divided into four main classes:

1. Liquid electrolytes (LE);
2. Solid polymer electrolytes (SPE);
3. Polymer gel electrolytes (PGE);
4. Nanocomposite polymer and gel electrolytes (NPEs).

In this review, primary attention is given to the consideration of ion transport processes in polymer gel electrolytes as the most promising for LIB. The review consists of two parts. First, the composition of electrolyte systems and their conductivity are considered. Second, the mechanisms of ion transport investigated by the nuclear magnetic resonance (NMR) technique are discussed. NMR is a very informative tool for characterizing both the structure and dynamics of processes in polymer gel electrolytes. In this paper, the NMR study of solid polymer electrolytes is not considered in detail since, in 2018, a review on this topic was already published in the same journal [13].

## 2. Electrolytes for Lithium-Ion Batteries, Compositions and Conductivity

### 2.1. Liquid Electrolytes

Liquid electrolytes for lithium power sources are still an object of research with the aim of their modification to achieve a more stable and safe operation of the whole electrochemical device, as shown in previous reviews [14,15,16].

Non-aqueous electrolytes are used in LIBs since lithium is very chemically reactive in aqueous solutions. In addition, aqueous electrolytes have a narrow window of electrochemical stability.

Predominantly, LIBs use a liquid electrolyte based on lithium salts in a mixture of aprotic organic solvents. The electrolyte must have sufficiently high conductivity and be resistant to oxidation and reduction.

The compounds LiPF_6_, LiClO_4_, LiBF_4_, LiSO_3_CF_3_ (LiTf), and LiN(SO_2_CF_3_)_2_ are used most frequently as lithium salts. Lithium bis(trifluoromethylsulfonyl)imide is abbreviated as LiTFSI.

The concentration of lithium salt in electrolytes at which the maximum conductivity is reached varies from 0.5 to 1.0 M for different compositions. In [17], an empirical formula was developed to determine the optimal salt concentration of the electrolyte at which the maximum conductivity is achieved. For example, for LiClO_4_ propylene carbonate solutions, this concentration is 0.5 M, while for the currently most used LiPF_6_ solutions in mixed carbonate solvents, this concentration is 1.0 mol.

The Handbook of Batteries [18] contains data on the conductivity of various mixed liquid electrolytes. It has also been shown that for electrolytes of composition 1 M LiPF_6_ in solvent mixtures, the conductivity is higher than electrolytes based on a single solvent.

The upper operating temperature of a liquid organic electrolyte is limited up to 60 °C, at which the redox decomposition of the lithium salt starts, or one of the cosolvents boils. When developing the electrolyte composition, it is necessary to carefully choose the electrolyte salt [19] and the type of solvent [14,20].

**Figure 1:**
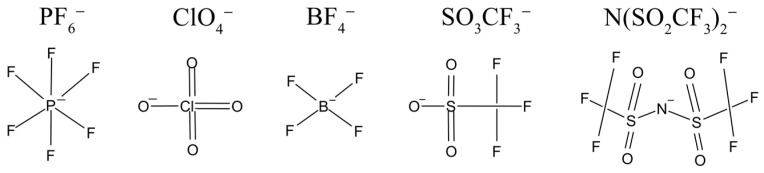
Lithium salt anions are commonly used in electrolytes of LIBs.

The structural formulas of anions of lithium salts used in electrolytes for LIB are shown in Figure 1.

The ion mobility increases with decreasing anion size in the series: LiBF_4_ > LiClO_4_ > LiPF_6_ > LiTf > LiTFSI.

These salts are dissolved in aprotic organic solvents with a high dielectric constant (ε), such as ethylene carbonate, EC (ε = 95.3 at 40 °C), propylene carbonate, PC (ε = 65.1), and γ-butyrolactone, GBL (ε = 39). However, because of their high viscosity and low melting temperature (EC melting temperature is about 40 °C), monosolvents are practically not used. In addition to these solvents, so-called thinners with low dielectric constant and low viscosity like dimethyl carbonate (DMC), ethyl methyl carbonate (EMC), diethyl carbonate (DEC), and 1,2-dimethyl ether (DME) are used. Their ε range is from 2.8 to 7.2.

The separator is usually polyethylene (PE) or polypropylene (PP), or a mixture thereof. A multilayer separator is made of layers from different polymers, but a disadvantage of such a separator is poor solvent retention.

Usually, LIBs with organic liquid electrolytes have disadvantages, such as flammability, leakage, and environmental toxicity, making their application difficult [21,22]. In this regard, replacing the liquid electrolyte with a polymer electrolyte seems to be a reliable solution to the aforementioned safety problems.

### 2.2. Solid Polymer Electrolytes

The first solid polymer electrolyte (SPE) for lithium power sources was proposed by Armand et al. in 1978 [23]. High-molecular-weight polyethylene oxide (PEO), –(C_2_H_4_O)_n_–, with a molecular weight of 500 000, was studied as a polymer matrix. The chain with 8 oxygen atoms had 1 Li^+^ ion. A hopping mechanism of conduction was assumed. The low conductivity of the SPE composition (PEO)_8_LiClO_4_, about 10^−8^ S/cm at 20 °C, was explained by the high degree of crystallinity of the polymer matrix from a high-molecular-weight linear polymer.

There are quite strict requirements for the selection of the SPE polymer matrix:

- The structure of the main or side chain of the polymer must contain heteroatoms with high basicity, capable of solvating Li^+^, thus contributing to the dissociation of the salt;
- Heteroatoms in the polymer chain should be located with such a periodicity that would facilitate the rapid transport of Li^+^ ions;
- To ensure the free movement of Li^+^ ions, the polymer must not be crystalline, and its glass transition temperature should be lower than the operating temperature of the power source;
- The polymer must be chemically and electrochemically stable with respect to the electrode materials and also be capable of forming mechanically strong films for assembling a chemical power source.

In addition to PEO, the following polymers meet these requirements: polyacrylonitrile (PAN), –CH_2_CH(–CN)–; polymethyl methacrylate (PMMA), –CH_2_C(–CH_3_)(–COOCH_3_)–; polyvinylidene fluoride (PVDF), –CH_2_CF_2_–; etc.

Network polymer electrolytes with a completely amorphous structure are very promising. Such a matrix can be formed by various polyether diacrylates, which, upon radical polymerization, can form a three-dimensional network of different degrees of cross-linking [24]. Among them, poly(ethylene glycol) diacrylate is of interest, where PEO units are located inside. This compound contains units of –CH_2_CH_2_O– at the edges of the C=C group to form joints.

In some works [25,26,27], polyethylene glycol methylacrylate (PEG MA) is used. Its formula is H_2_C=CHCO_2_(CH_2_CH_2_O)_n_CH_3_. It has a C=C reaction bond on one side only. Therefore, it is used as a copolymer with another oligomer.

The compositions and conductivity of some solid polymer electrolytes are shown in Table 1.

Despite the advantages of SPE, their use is severely limited because the rigid structure of the solid polymer electrolyte demonstrates insufficient ionic conductivity (Table 1).

### 2.3. Polymer Gel Electrolytes

**Figure 2:**
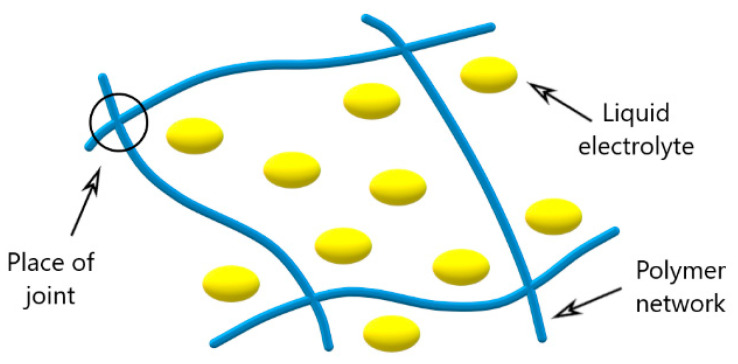
Scheme of cross-linkage of the polymer gel electrolyte.

Polymer gel electrolytes consist of a polymer matrix containing solvents that solvate the ions. Figure 2 shows a schematic representation of the PGE.

Gels can be divided into two categories according to their method of preparation:

1. Physical gels are formed when a liquid electrolyte is placed in a polymer matrix without the formation of chemical bonds between the polymer and solvent, for example, a liquid electrolyte solution in PMMA or PVDF;
2. Chemical gels are obtained by chemical cross-linking of a polymer matrix in a liquid organic electrolyte, for example, PEG DA.

The lithium salt is responsible for conduction in the polymer matrix, while the polymer holds the electrolyte to provide mechanical strength. Compared to SPEs, PGEs show higher ionic conductivity, a wide electrochemical stability window, and good compatibility with electrodes due to their increased ionic mobility. PGEs have both cohesive properties of solids and diffusion properties of liquids, as well as good mechanical strength. In PGE, the role of the polymer is to form a solid matrix that supports the migration of ions in solvents, where a conductivity value of about 10^−3^ S/cm at room temperature can be achieved. Here, the electrolyte can act as a plasticizer, reducing the glass transition temperature, which results in higher ionic conductivity.

If, for SPE, the main matrix is PEO, then the more common polymer matrix for impregnation with liquid electrolyte is poly(vinylidene fluoride-*co*-hexafluoropropylene), –(CH_2_–CF_2_)_x_–(CF_2_–CF(–CF_3_))_y_–. The PVDF–HFP copolymer acts as a separation material that swells in an organic liquid electrolyte.

Although high ionic conductivity is an important advantage of PGE over SPE, other characteristics must also be taken into account for the practical use of PGE:

(1) The ability to retain the liquid phase of the electrolyte;
(2) Mechanical strength;
(3) Conductivity in a wide temperature range.

For example, for PGE, solvent losses are often observed due to leakage or evaporation, and, as a result, the cell resistance increases and the contact between the electrodes are broken.

Table 2 lists the composition and characteristics of some polymer gel electrolytes.

Table 2 shows that various polymer gel systems exist. Various polymers, salts, and solvents, including ionic liquids, are used by different researchers.

The safety of gel electrolytes is determined by the degree of fluid retention of the polymer.

The electrochemical stability of the liquid electrolyte at the interface with the electrode is much lower than that of a solid polymer electrolyte. Gel electrolytes have an intermediate composition. Therefore, the stronger the contact between the liquid phase and the electrode, the lower the decomposition potential of the gel electrolyte.

The highest degree of retention of the liquid phase is observed in the mesh matrices formed by chemical crosslinking (PEG-DA, PEDA). PEDA [53,54] is a product of the anionic polymerization of 2-hydroxyethyl acrylate and 4,4′-dicyclohexylmethane diisocyanate. Polar NHCO groups, when introduced into the PEDA chains, favor stronger retention of a polar electrolyte and higher stability of a polymer gel electrolyte.

**Figure 3:**
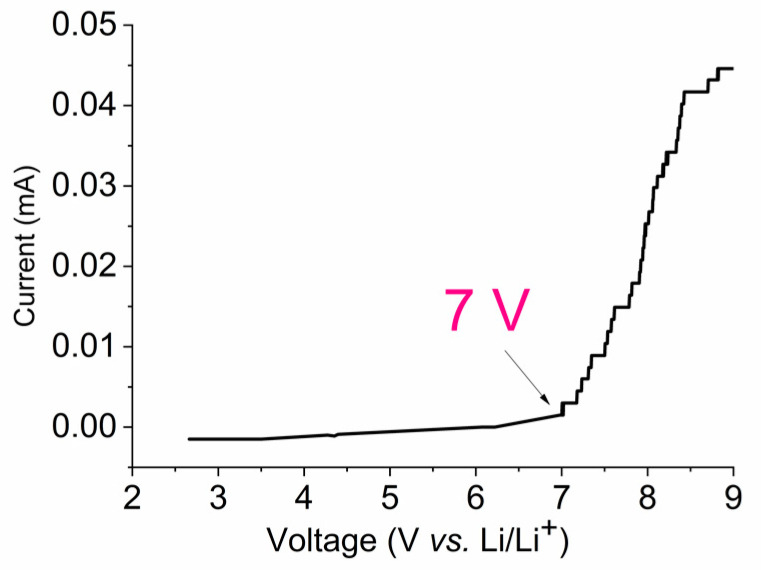
Voltammogram of Li/PGE/Stainless Steel at 2 mV/s rate scanning with PEDA:EC (1:1 *w*/*w*) and LiClO_4_ 7.5 wt %.

Figure 3 shows the voltammogram of the Li/PE/NS cell for the electrolyte composition PEDA–LiClO_4_–EC. The upper window of electrochemical stability is almost 7 V vs. Li^+^/Li [62].

In the membranes based on PVDF-HFP, the retention of the liquid phase is much lower, and, therefore, the solvent has more contact with the electrode material. The electrochemical stability of PVDF-HFP-based electrolytes is 4–5 V. Therefore, works on modifying these membranes are permanently being developed.

Thus, in [45], the authors fabricated a three-layer polymer membrane by placing PVDF on the outer sides. The inner layer was made of methylcellulose, an environmentally friendly and cheap product. The outer layers of PVDF are porous, resulting in a high electrolyte uptake. The resulting Li^+^ ion transport number was higher than that of the pure methylcellulose-based electrolyte.

Unfortunately, along with a high conductivity of about 10^−3^ S/cm at 20 °C, PGEs have a significant drawback: instability because of a gradual change in the concentration of the organic solvent, which is part of the PGEs, and poor mechanical strength. One of the solutions to these problems is the introduction of nanodispersed additives into the PGE composition and the transition to the so-called class of nanocomposite polymer electrolytes (NPEs).

### 2.4. Nanocomposite Polymer Electrolytes

Nanocomposite polymer electrolytes for lithium power sources are a promising class of electrolytes for fully solid lithium and lithium-ion batteries, which have increased operational safety [10,11].

Initially, nanoadditives of oxides Al_2_O_3_, SiO_2_, TiO_2_, etc. were introduced into the SPE matrix to reduce the crystallinity of the polymer and remove traces of moisture because these substances are desiccants.

However, this approach could not radically solve the problem of low conductivity. Nanoparticles then began to be introduced into the composition of PGEs [63]. These works appeared more than 15 years ago. Thus, a new class emerged: nanocomposite polymer gel electrolytes, which are a compromise option that combines the liquid phase conductivity of PGEs and the improved mechanical properties of solid NPEs.

In addition to increasing conductivity and mechanical strength, nanoparticles can reduce the resistance at the electrode/electrolyte interface by shortening the contact area of the liquid phase with the electrode surface. Consequently, the window of electrochemical stability of the electrolyte can expand.

The same polymers, salts, and solvents can be used as the NPE matrix for both SPEs and PGEs, where these solvents act as polymer plasticizers.

For example, Table 3 shows the compositions and conductivity of some recently developed NPEs. Electrolytes nos. 1–7 are solid NPEs. Their composition is a polymer–salt nanoparticle with a conductivity of 10^−4^–10^−6^ S/cm. The other NPE compositions have an additional liquid phase.

Table 3 shows that the majority of the gel NPEs have a conductivity of about 10^−3^ S/cm at *T*_room_, and the best particles for filling are TiO_2_ and SiO_2_. These nanoparticles improve the mechanical strength of the gels and contribute to the conductivity of lithium and sodium ions. As shown in [64,65], SiO_2_ nanoparticles are involved in the dissociation of the electrolyte salt and, hence, the number of charge carriers increases.

In addition to NPEs, there are also hybrid polymer electrolytes [9,66,67], where the role of nanoparticles is played by other particles, for example, octavinyloctasilsesquioxane (POSS) [68,69] or lithium polyvinyl alcohol oxalate borate [70].

## 3. NMR Study of Polymer Electrolytes

The synthesis of new polymer electrolytes with desired properties and their efficient use requires establishing a relationship between the structure of the polymer matrix, the features of ion solvation, the structure of transport channels, and the parameters of electro mass transfer, in particular, ionic conductivity and translational ionic and molecular mobility. The impedance spectroscopy method allows one to measure the integral ionic conductivity but provides no information about charge carriers. An important advantage of NMR techniques, especially pulsed field gradient NMR, is the ability to selectively measure the mobilities and self-diffusion coefficients for all ions and molecules involved in charge transfer, for example, solvents (^1^H and ^13^C NMR), lithium cations (^7^Li NMR), and anions (^11^B, ^19^F NMR).

In this review, we consider works studying polymer electrolytes of all three classes by NMR methods.

This review summarizes the results of studies carried out by NMR spectroscopy, NMR relaxation, and pulsed magnetic field gradient NMR. Such an analysis makes it possible to establish a number of fundamental regularities of ionic and molecular transport in polymer electrolytes at the molecular level.

NMR is widely applied for polymer electrolytes. The main direction of investigations is lithium cation surroundings, local and macroscopic mobilities of Li^+^ and F^−^-containing fragments, and electrolyte composition–ion transport correlations. Modern and complicated NMR techniques are employed, especially solid-state high resolution magic angle spinning (MAS) NMR, pulsed-field gradient NMR, and NMR spin relaxation [13,29,32,33,34,37,39,40,42,47,49,50,51,52,53,54,56,57,58,59,60,61,65,72,73,76,77,80,92,95,96,97,98,99,100,101,102,103,104,105,106,107,108,109,110,111,112,113,114,115,116,117,118,119,120,121,122,123,124,125,126,127,128].

### 3.1. NMR in Solid Polymer Electrolytes

In this paper, the NMR study of solid polymer electrolytes is not considered in detail since, in 2018, a review on this topic was already published in the same journal [13].

In this chapter, NMR studies of SPE are briefly reviewed.

Solid polymer electrolytes of a polymer–salt composition are studied by different NMR methods. The review [108] is devoted to solid-state NMR spectroscopy for the characterization of the molecular structure and dynamics in solid polymers and hybrid electrolytes. In the authors’ opinion, the NMR techniques may be very valuable for materials of lithium- and sodium-based batteries. There are solid-state NMR, pulse field gradient NMR, electrophoretic NMR, variable temperature *T*_1_ relaxation, *T*_2_ relaxation and line width analysis, exchange spectroscopy, cross-polarization, rotational echo double resonance, and isotope enrichment applications.

Let us enumerate the most interesting NMR results in other works.

^7^Li and ^19^F NMR provide information related to local ion dynamics and diffusion coefficients in poly(ethylene oxide) polymer electrolytes that correlate with the conductivity behavior [116].

Two different Li^+^ positions in the PEO/Li^+^ complex structures were observed by solid-state NMR. The 2D ^7^Li exchange NMR showed the exchange process between the different Li^+^ species. The exchange dynamics of the Li^+^ ions provide the molecular mechanism of the Li^+^ transportation on the surface of PEO crystal lamella, which correlates with the ionic conduction mechanism [51].

MAS NMR ^7^Li in poly(ethylene oxide carbonate) with methacrylic monomer showed that the Li^+^ coordination surrounding depends on the LiTFSI concentration [29].

MAS NMR, PFG NMR, NMR relaxation, and two-dimensional exchange 2D EXSY NMR were applied to LISICON-, NASICON-, and Garnet-type lithium-ion conductors. Two ion diffusion pathways for Li^+^ cation were observed [103].

Comb-like solid polymer electrolytes were studied using cross-polarization ^13^C-^1^H solid-state NMR. A correlation between ^13^C solid-state NMR measurements and phase segregation was determined. ^7^Li NMR spectroscopy was used to characterize the mobility of lithium ions. It was suggested that lithium ions interact with the PEG-MA pendant groups [107].

^1^H, ^7^Li, and ^19^F NMR were applied to selectively investigate polymer, cation, and anion dynamics in mixing non-entangled poly(propylene glycol) (PPG) with LiClO_4_ or LiTFSI polymer electrolytes of various length and time scales and over broad temperature ranges. It was observed by static field gradient diffusometry that the long-range motion of all components slows down with increasing salt concentration. Cations are less mobile compared to anions. The Arrhenius temperature dependence does not approximate the ionic diffusion coefficients. It was shown by spin-lattice and spin-spin relaxation that the local lithium and polymer dynamics depend on the salt content. The segmental motion is bimodal for intermediate salt concentrations because two regions (salt-rich and salt-depleted) coexist. Lithium-ion transport is strongly related to polymer segmental motion. The reorganization of polymer chains and lithium-ion transport is controlled by the Rouse dynamics [112].

Fast lithium-ion transport in the crystalline polymer electrolytes was found. The polymer electrolyte composition CD-PEOn/Li^+^ (*n* = 12, 40) was prepared by self-assembly of α-cyclodextrin (CD), polyethylene oxide, and Li+ salts. The solid-state NMR method combined with the X-ray diffraction technique reveals the following structural features: (a) ordered long-range pathways are formed by CD associates for Li+ ion transport; (b) a sequence of the PEO chains in the tunnels appeared, attenuating the coordination of Li+ significantly by the EO segments [114]. As revealed from ^1^H and ^7^Li NMR, the polymer electrolyte based on low-molecular-weight PEOs and cyclodextrin exhibits extremely fast Li+ ion transport [34].

### 3.2. NMR of Liquid Electrolytes

Some recent NMR data in liquid electrolytes are found in [42,95,98,100,102,120,121].

NMR techniques are used to study ion transport in liquid electrolytes and assess the composition of the solvate environment of ions. Therefore, in [95], the dissociation of LiPF_6_ in the nonaqueous cyclic propylene carbonate was revealed by ^7^Li NMR Overhauser enhancement spectroscopy. The coordination number of the solvent and average sizes of solvated and ion-paired clusters were estimated from the PFG NMR data.

Self-diffusion of ^1^H, ^7^Li, and ^19^F was studied in the electrolytes containing LiTFSI salt dissolved in *tert*-butyl methyl ether (MTBE) or tetrahydrofuran (THF) and propylene carbonate (PC), depending on the concentration. At the concentration 1:16 LiTFSI:MTBE and 1:16 LiTFSI:THF, the self-diffusion coefficients decrease with increasing temperature due to ion aggregation. Ionic conductivities increase with increasing temperature [129].

In addition, to characterize the liquid electrolyte itself, its mobility in the pores of separators (PVDF [42] or PE [98]) or electrodes [100] may also be studied by NMR. Self-diffusion coefficients of lithium cations, BF_4_ anions, and solvent molecules were measured by PFG ^7^Li, ^19^F and ^1^H NMR in LiBF_4_, propylene carbonate, and PVDF, respectively [42]. Restricted diffusion of a LiPF_6_ electrolyte solution in porous polyethylene was observed by PFG ^1^H, ^19^F, and ^7^Li NMR, and some structural parameters were estimated [98]. MAS and PFG NMR were used to measure the self-diffusion coefficients of LiPF_6_ in EC/DMC for electrode pore characterization [100].

The study of liquid electrolytes by high-resolution NMR spectroscopy is considered in more detail.

Solutions of LiBF_4_ and LiClO_4_ in ethylene carbonate (EC) were studied by high-resolution ^1^H, ^7^Li, ^11^B, ^13^C, ^17^O, ^35^Cl NMR spectroscopy as model systems [120,121].

**Figure 4:**
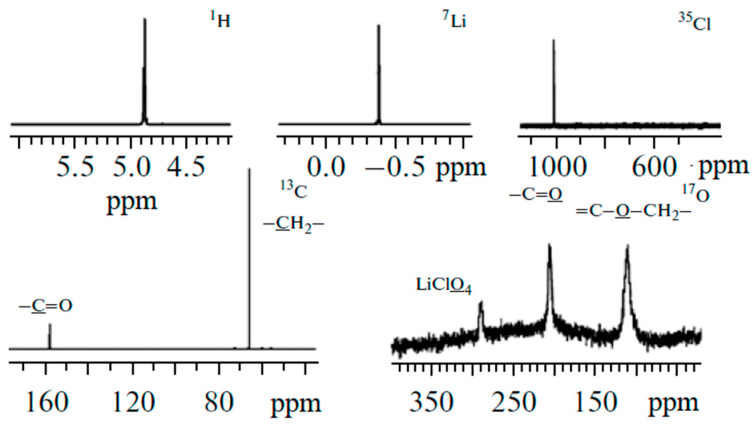
^1^H, ^7^Li, ^35^Cl, ^13^C, and ^17^O NMR spectra of LiClO_4_–ethylene carbonate solutions [120].

**Figure 5:**
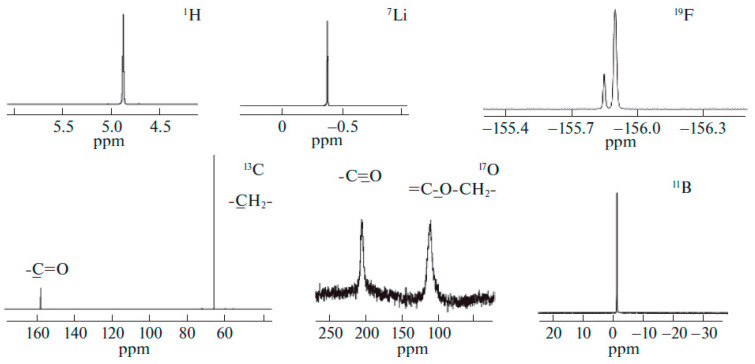
Experimental ^1^H, ^7^Li, ^19^F, ^13^C, ^17^O, and ^11^B NMR spectra of a 0.66 molality solution of LiBF_4_ in ethylene carbonate [121].

The corresponding NMR spectra are shown in Figure 4 and Figure 5.

**Figure 6:**
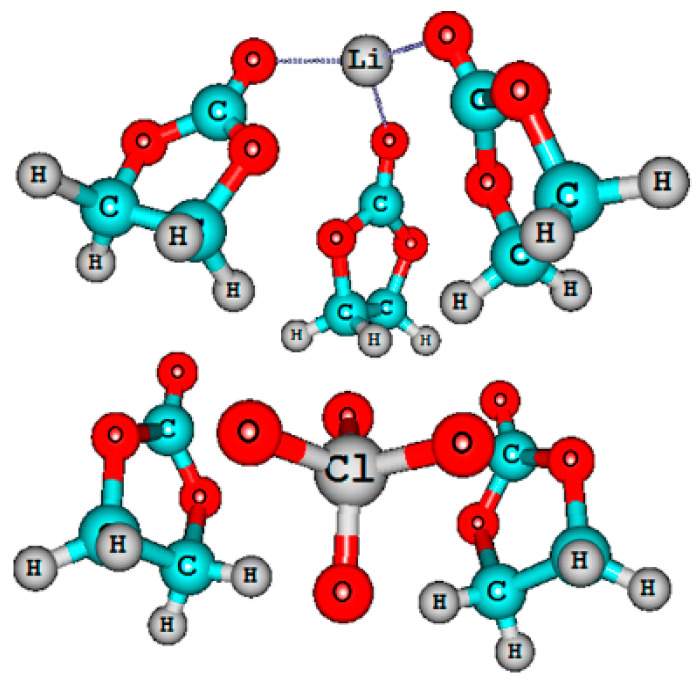
Molecular structures of ionic pair in a 3 molarity LiClO_4_–ethylene carbonate solution [120].

**Figure 7:**
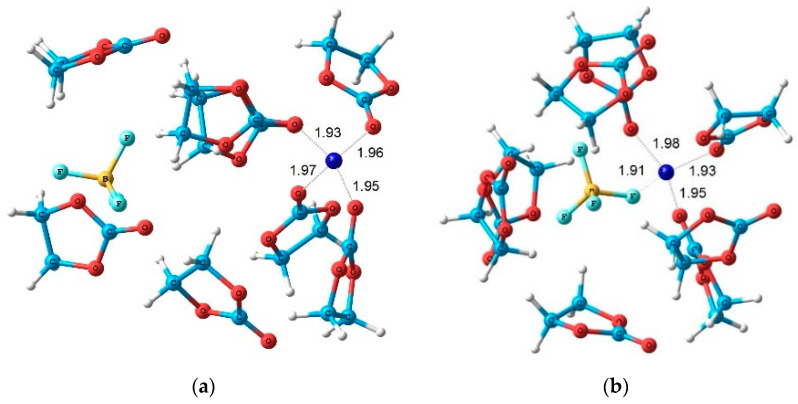
Theoretical structures of (**a**) Li^+^–4ECs and BF_4_ –4ECs solvate-separated ion pair and (**b**) LiBF_4_–8ECs contact ion pair. EC: ethylene carbonate. Interatomic distances are given in angstrom [121].

The quantum-chemical calculations of nuclear chemical shifts were performed. The structure models of cation-anion-solvent complexes depending on concentration were proposed on the basis of a comparison of the experimental and calculated chemical shifts. Some examples of the complex structure are shown in Figure 6 and Figure 7.

An analysis of the nuclear chemical shift dependences on the concentration made it possible to calculate the degree of dissociation for LiBF_4_ in EC [121]. At a low concentration, Li^+^ ions are surrounded by the solvate shell only and isolated. Ion solvate-separated and contact ion pairs are formed when the concentration increases [121].

### 3.3. NMR in Polymer Gel Electrolytes

The results of the NMR study of gel electrolytes are given in [39,40,47,49,50,53,54,56,57,80,119,122,124,125,126,130].

Gel-polymer electrolytes are studied mainly by PFG NMR and MAS NMR. In [80], the lithium transport number, ionic association degree, and self-diffusion coefficients were measured in a polyethylene glycol dimethyl ether (MW 500) dissolved in a LiCF_3_SO_3_ electrolyte.

In addition to the experimental methods of NMR and AC impedance spectroscopy, the method of molecular dynamics (MD) is also used. MD is mainly applied to solid polymer [131] or liquid [132] electrolytes. The gel polymer electrolytes are considered in only one paper [32].

Molecular dynamics and density functional theory simulations and ^7^Li NMR in crosslinked poly(tetrahydrofuran) (xPTHF) show a decrease in the content of oxygen atoms in the xPTHF backbone, which leads to loosening of O^−^Li^+^ coordination that enhances ion transport [32]. Gel polymer electrolytes based on PVDF-HFP-containing propylene carbonate, isobutyronitrile (IBN), and trimethyl acetonitrile (TMAN) solvent blend electrolytes were developed. Electrochemical impedance spectroscopy, PFG NMR, and relative permittivity determination revealed remarkable ion-conducting properties of IBN and TMAN solvents [39]. Spin-lattice relaxation times (*T*_1_) and self-diffusion coefficients of ^7^Li and ^19^F were measured in the gel polymer electrolytes based on a polyacrylonitrile elastomer. This study shows that a high acrylonitrile content in the polymer and a solvent with a moderate donor number increase the Li^+^ mobility [56].

A very important question is Li^+^ cation surroundings. Unfortunately, the ^7^Li chemical shift is varied within a range of only 1–2 ppm, which is comparable with the NMR line width even in the MAS spectra. Some results of ^7^Li NMR spectra deconvolution are discussed below.

Gel polymer electrolytes based on polyester diacrylate PEDA and ethylene carbonate were investigated. The polymer electrolyte compositions are shown in Table 4 [53].

**Figure 8:**
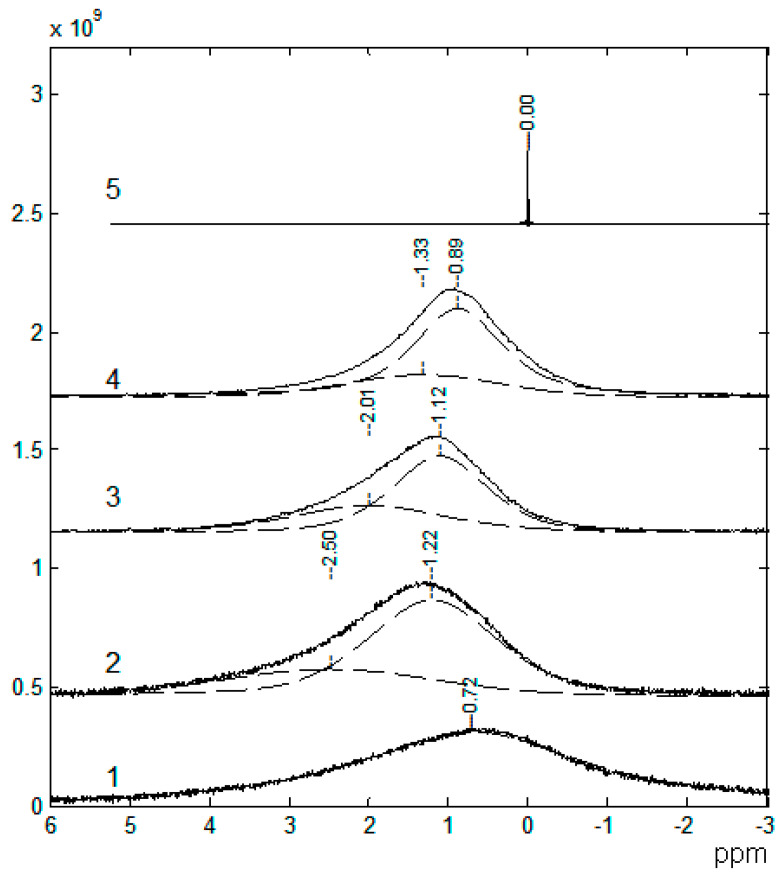
^7^Li NMR spectra: 1, sample no. 1; 2, sample no. 2; 3, sample no. 5; 4, sample no. 9; 5, 1 M LiClO_4_ solution in ethylene carbonate [53].

The ^7^Li NMR spectra are asymmetric and can be decomposed into two singlet lines that differ in width and chemical shift (Figure 8). The ^7^Li NMR spectrum of the polymer without solvent is a wide singlet line. In the polymer-solvent system, two NMR lines are observed, the chemical shifts and width of which depend on the EC content. It was concluded that the narrow line was due to mobile Li^+^ coordinated by EC molecules, and the wide line belonged to Li^+^ that interacted with the polymer matrix.

Gel electrolytes with the addition of tetraethoxysilane were studied [123]. The MAS ^7^Li spectra were recorded in the temperature range of −140 °C to 80 °C. At low temperatures, Li^+^ forms a complex with the polymer. At high temperatures, Li^+^ is coordinated by solvent molecules.

**Figure 9:**
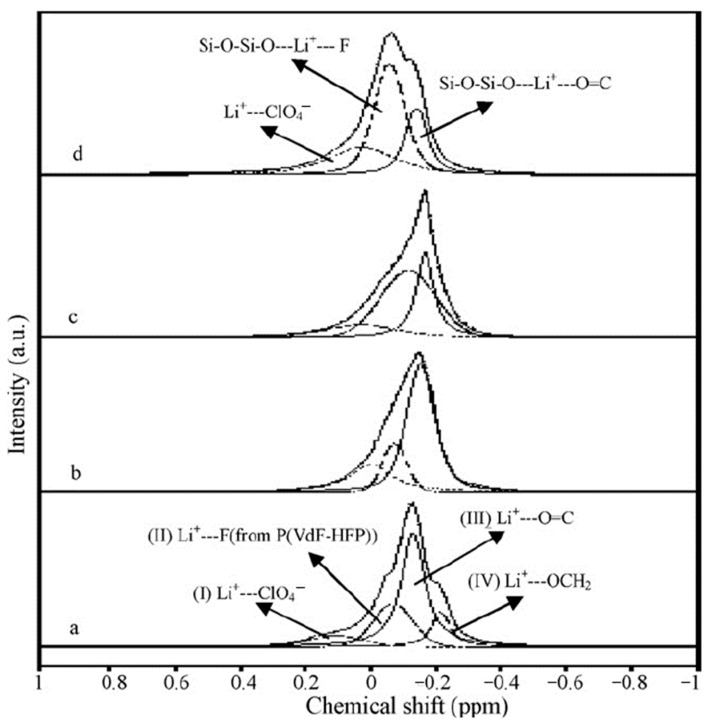
MAS ^7^Li spectra of (PVDF-HFP)-(PC + EC)-LiClO_4_-SAG. Component contents (wt %): 20:70:10:0 (**a**), 20:69:10:1 (**b**), 20:66:10:4 (**c**), 20:64:10:6 (**d**). Spinning frequency is 2 kHz [122].

The ^7^Li NMR spectra of the composite electrolyte gel, PVDF-HFP, with the addition of silicate aerogel (SAG) and ethylene carbonate and propylene carbonate as solvents at different contents of aerogel and LiClO_4_ are shown in Figure 9 [122].

Four spectrum components were revealed: the peak at 0.104 ppm in gel electrolyte without SAG shows ion pairs and aggregates, the peak at 0.07 ppm belongs to Li^+^ interacting with PVDF-HFP fluorine atoms, and the peaks at 0.13 and 0.21 ppm are Li^+^ coordinated by PC and EC, respectively [124,125]. The shape and intensity of individual component lines are changed with SAG addition (12 wt %). This indicates the interaction of SAG and Li^+^ in the solvent phase and a decrease in the ion pair fraction, which is consistent with the conductivity data. Therefore, it may be concluded that Li^+^ cations interact with SAG, solvent, and PVDF-HPF. These interactions could be favored by the formation of transport conditions for the fast movement of Li^+^ cations over the SAG particles’ surface.

**Figure 10:**
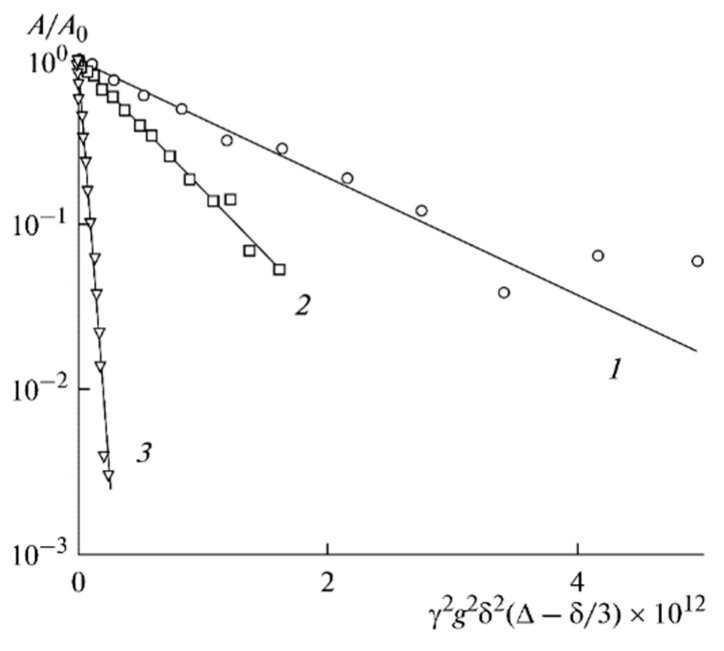
Dependences of the ^7^Li spin-echo signal amplitudes on the square of magnetic field gradient amplitude (diffusion decay) for PGE samples: (**1**) no. 2, (**2**) no. 5, and (**3**) no. 9 [53].

The ^7^Li diffusion decay is approximated by Equation (1) [126], Figure 10.

(1) $$A\left(g\right)=A\left(0\right)\exp\left(-\gamma^{2}g^{2}\delta^{2}t_{d}D_{s}\right),$$

where *A* is the echo signal intensity at the magnetic field gradient pulse with the amplitude *g*, *A*_0_ is the echo signal intensity without gradient pulse, *δ* is the duration of the magnetic field gradient pulse, Δ is the interval between the gradient pulses, *γ* is the nuclear gyromagnetic ratio, and *D_s_* is the self-diffusion coefficient.

**Figure 11:**
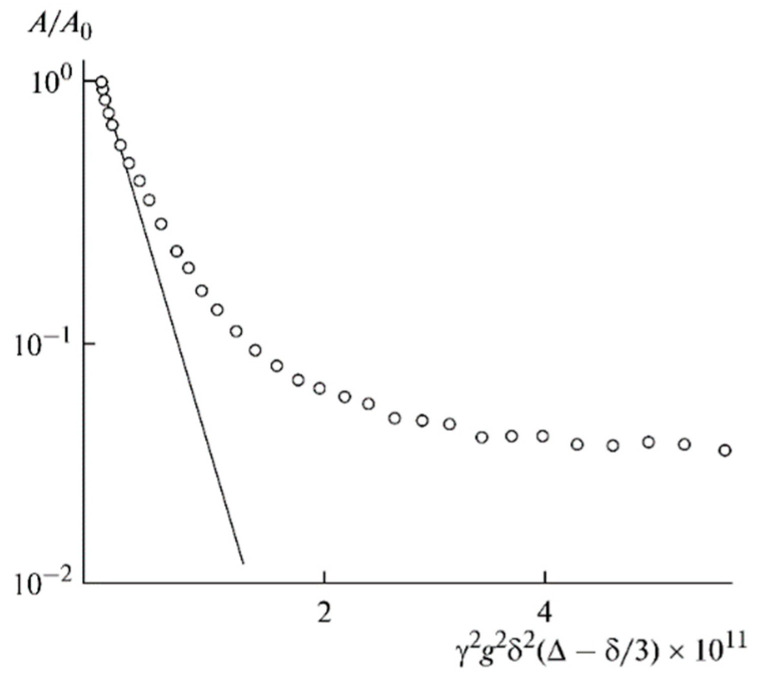
Example of ^1^H diffusion decay in PGE sample no. 7 [53].

As shown in Figure 10, the translational motion of Li^+^ cations is characterized by the self-diffusion coefficient only regardless of the EC content. The diffusion decay shape of ^1^H is more complicated (Figure 11) and can be decomposed into three exponential components described by Equation (2) [126]. The self-diffusion coefficient of H atoms can also be characterized by the average coefficient.

(2) $$A\left(g\right)=p_{1}\exp\left(-\gamma^{2}g^{2}\delta^{2}t_{d}D_{s1}\right)+p_{2}\exp\left(-\gamma^{2}g^{2}\delta^{2}t_{d}D_{s2}\right)+p_{3}\exp\left(-\gamma^{2}g^{2}\delta^{2}t_{d}D_{s3}\right)$$

The ionic conductivities *σ*_NMR_ were calculated from Li^+^ self-diffusion coefficients using the Nernst–Einstein Equation (3).

(3) $$\sigma =Ne^{2}\frac{D}{kT}$$

where *N* is the number of Li^+^ ions per unit volume, *e* is the electron elemental charge, *k* is the Boltzmann constant, *T* is the absolute temperature, and *D*_Li+_ is the Li^+^ self-diffusion coefficient.

**Figure 12:**
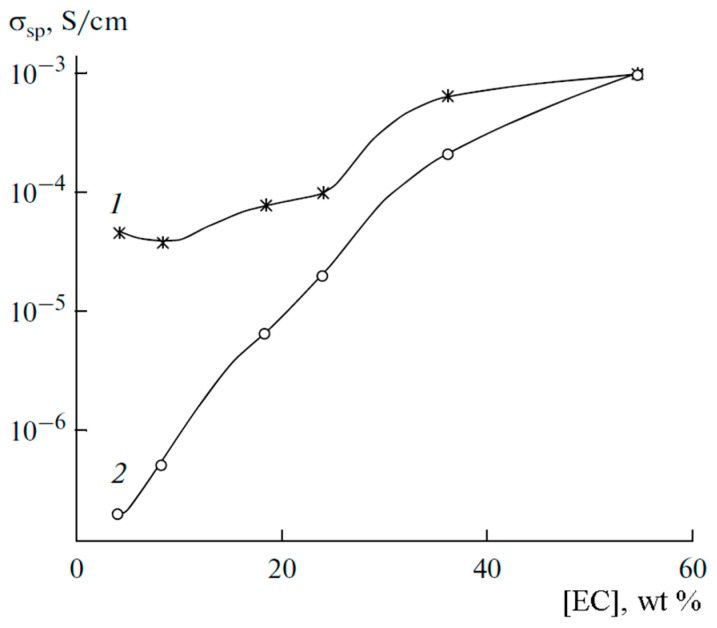
Dependences of the conductivity on the solvent content in PGE at 30 °C: (**1**) conductivity calculated by Equation (3), and (**2**) conductivity measured by impedancemetry [53].

Figure 12 shows the calculated conductivity *σ*_NMR_ and experimental conductivity *σ*_exp_ measured by the impedance spectroscopy dependences on ethylene carbonate content.

Conductivities increase with a decrease in the polymer concentration. At low EC contents, the calculated conductivity exceeds the measured values by 1–2 orders of magnitude, but the calculated and measured values are equal at the maximum EC.

This fact is explained by the fact that in Equation (3), *N* is the number of Li^+^ cations supposing that all perchlorate molecules are dissociated, which occurs at a high EC content only (experimental and calculated conductivities are equal), whereas at low EC LiClO_4_ molecules are incompletely dissociated. The degree of dissociation *α* can be calculated as the ratio *α* = *σ*_sp_/*σ*_NMR_.

**Figure 13:**
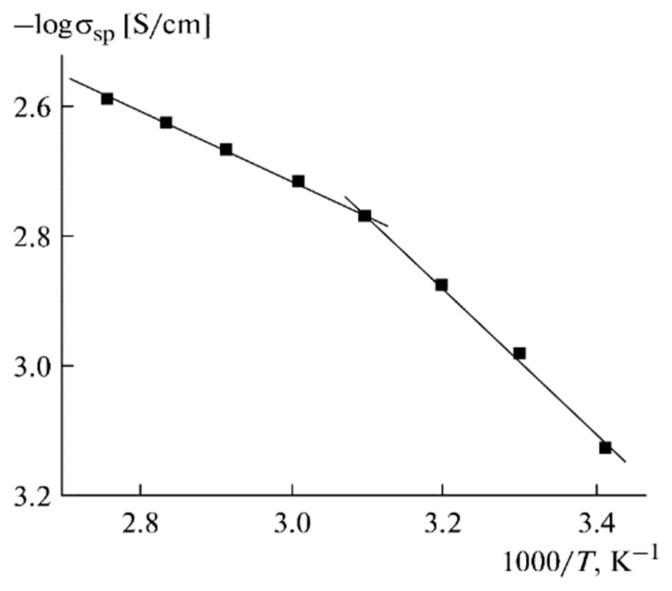
Temperature dependence of the specific ionic conductivity for PGE no. 9 [53].

**Figure 14:**
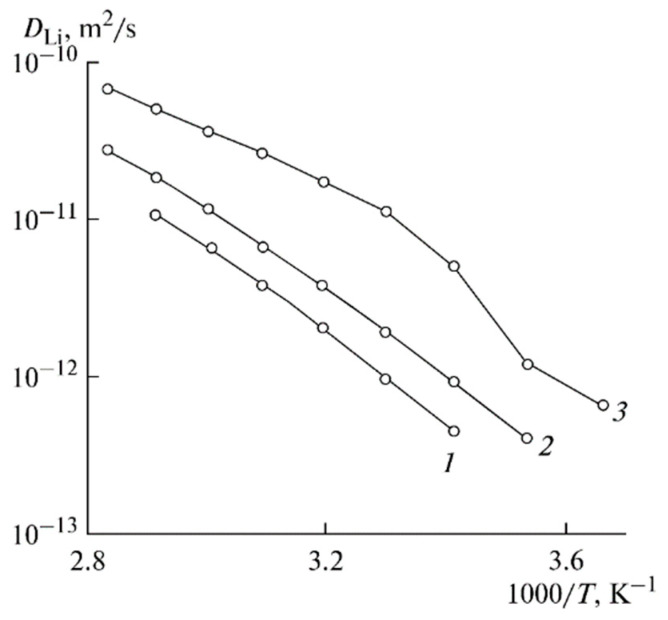
Temperature dependences of the Li^+^ self-diffusion coefficients for PGE no. (**1**) 2, (**2**) 5, and (**3**) 8 [53].

The temperature dependences of the ionic conductivity and lithium self-diffusion coefficient are shown in Figure 13 and Figure 14, respectively.

The Arrhenius law approximates these dependences. The temperature dependences of the ionic conductivity are approximated by two linear regions (Figure 13). The self-diffusion coefficients, ionic conductivities, and activation energies are listed in Table 5.

**Figure 15:**
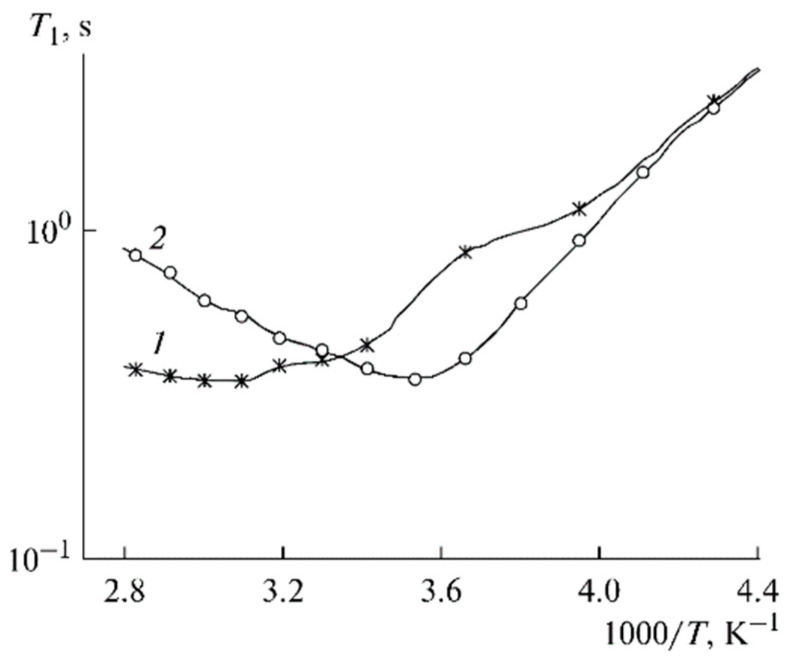
Temperature dependences of the ^7^Li spin-lattice relaxation time *T*_1_ for PGE nos. (**1**) 2 and (**2**) 9 [53].

Local ionic and molecular motion correlation times can be calculated from spin-relaxation of the ^7^Li and ^1^H temperature dependences [126]. The temperature dependences of the ^7^Li spin-lattice relaxation time *T*_1_ for PGE nos. (1) 2 and (2) 9 are shown in Figure 15. These dependences show minimum correspondence to the conditions *ωτ*_c_ ≈ 1, where *ω* is the NMR frequency, and *τ*_c_ is the correlation time. As shown above, the correlation time *τ*_c_ is, crudely, an elementary translational jump for a distance comparable with the size of a solvated cation.

**Figure 16:**
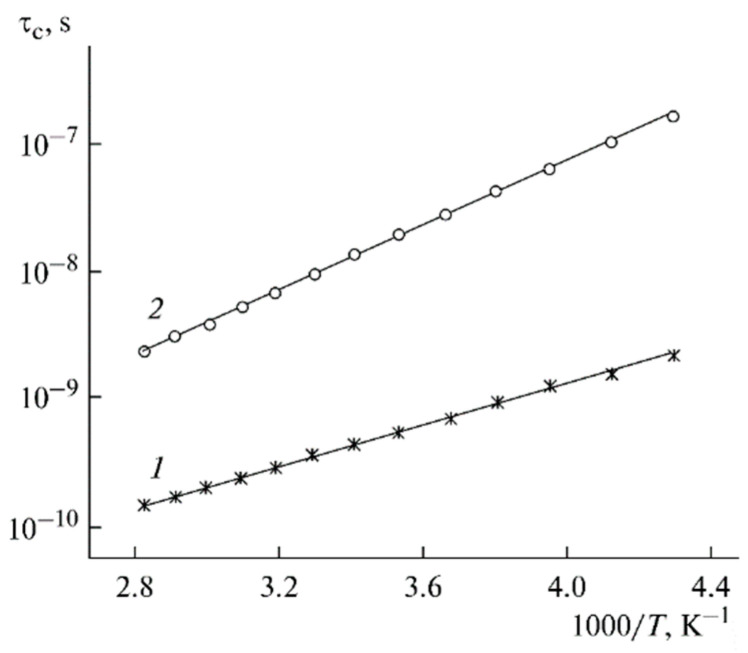
Temperature dependences of the correlation times of ^7^Li^+^ in PGE nos. (**1**) 2 and (**2**) 9 [53].

The temperature dependences of the correlation times are shown in Figure 16.

Assuming that the size of a solvated cation *l* is about 0.5 nm from the Einstein relationship (*D*_Li_ = *l*^2^/6*τ*_c_), the calculated values are 1.5 × 10^−6^ cm^2^/s for the sample with the maximum solvent content (no. 9) and 4 × 10^−8^ cm^2^/s for sample no. 2 (minimum EC content).

As follows from Table 5, these values differ by one order of magnitude. This is an acceptable agreement because the estimation of the correlation time is very crude. It may be assumed that the lithium cation macroscopic transfer is controlled by translational jumps over the distances compared with the sizes of solvated cationic complexes.

**Figure 17:**
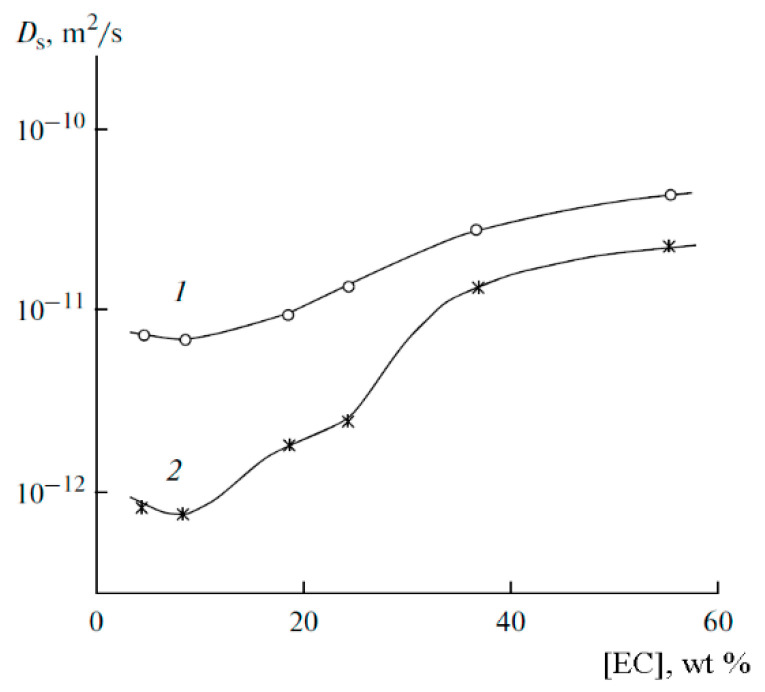
Dependences of the average self-diffusion coefficient of ^1^H (**1**) and self-diffusion coefficient of ^7^Li^+^ (**2**) on the solvent concentration [53].

The dependences of the self-diffusion coefficients of ^7^Li and ^1^H are symbate (Figure 17).

**Figure 18:**
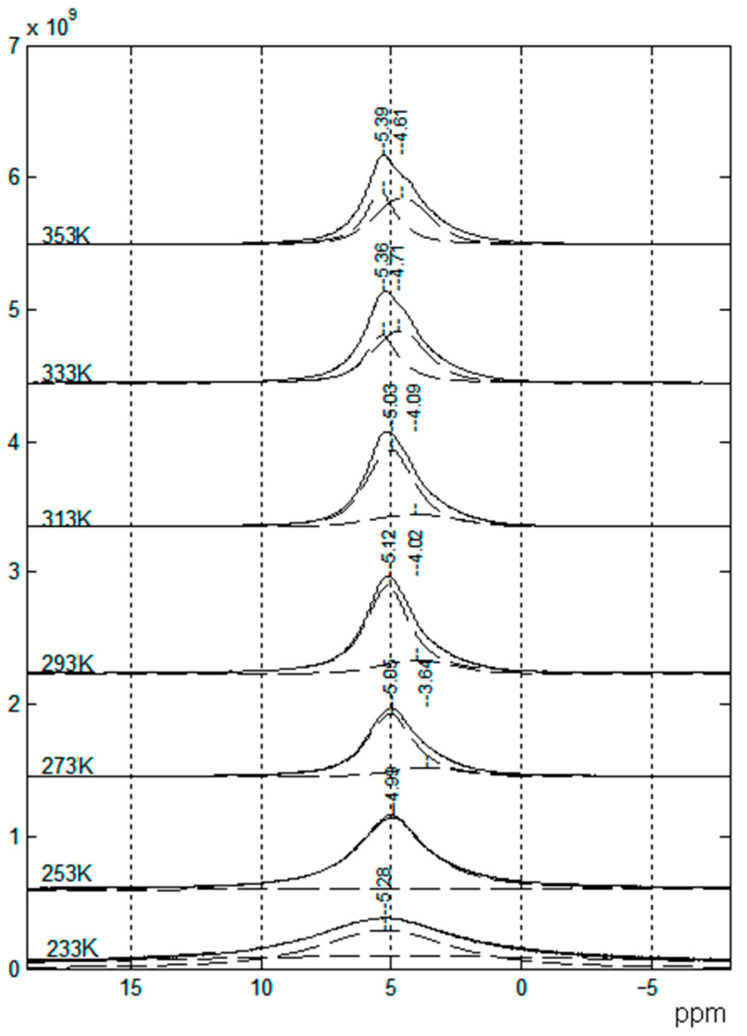
^1^H NMR spectra of SPE no. 9 at different temperatures [53].

Similarly, the ^7^Li and ^1^H spectra contain at least two components (Figure 18), which may be attributed to protons of PEDA and solvent molecules.

**Figure 19:**
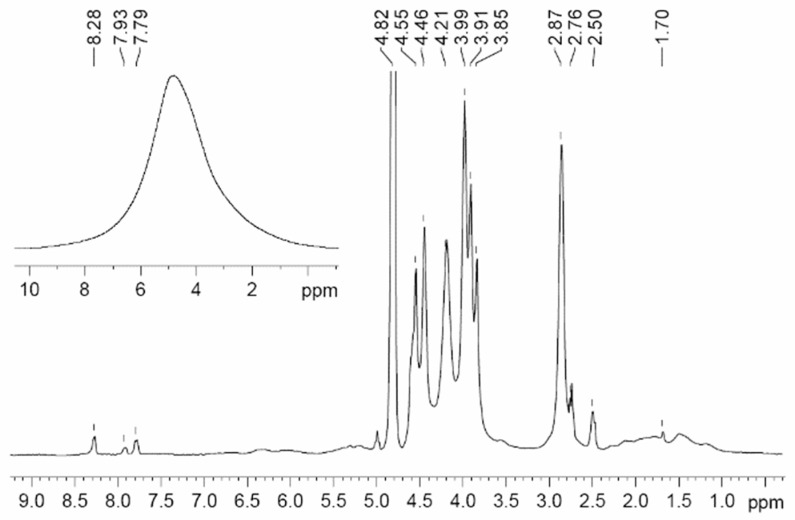
^1^H MAS NMR spectrum of PGE containing 54.8 wt % EC (no. 9, Table 1). Chemical shifts were measured relative to ethylene carbonate CH_2_ group protons (4.82 ppm relative to TMS). The sample spinning rate is 5 kHz. The ^1^H NMR spectrum without magic angle spinning is shown in the insert [54]. (*δ*): 4.82 (br.s, CH_2_, EC); 4.46, 2.76 (both br.s, CH_2_ and CH_2_CO, 2-hydroxyethyl acrylate dimer); 3.99, 2.87 (both br.s, CH_2_O and CH_2_CO, polymeric units of PEDA); 1–2.50 (br.m, CH and CH_2_, cyclic groups of PEDA); 7.79, 8.28, 7.93 (all br.s, NH, H_2_C=CH, PEDA); 4.20 (br.s, OH, HEA); 3.91, 3.85, 2.50 (br.s, CH_2_, HEA).

The NMR spectra of polymer electrolytes are usually not resolved (insertion spectrum in Figure 19). Therefore, a high resolution in the solid magic angle spinning (MAS) technique may be very efficient (well-resolved spectrum in Figure 19). Valuable information concerning polymer electrolyte chemical structure and composition can be obtained from the MAS ^1^H and ^13^C NMR spectra. The ^1^H NMR MAS spectrum of PGE containing 54.8% EC (no. 9, Table 4) is shown in Figure 19.

A decrease in the solvent concentration is accompanied by broadening and weakening of the line of methylene protons of EC (4.82 ppm). Thus, the MAS NMR spectra confirm the chemical structure and composition of the polymer electrolyte.

**Figure 20:**
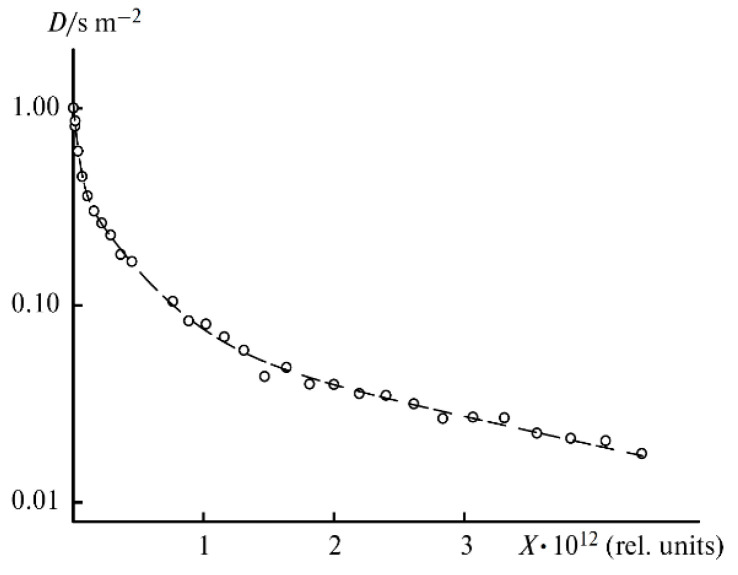
^1^H diffusion decay of PGE containing 12% EC wt % (sample 4, Table 1) and its approximation (dashes), *X* = *γ*^2^*g*^2^*δ*^2^(Δ—*δ*/3) [54].

**Figure 21:**
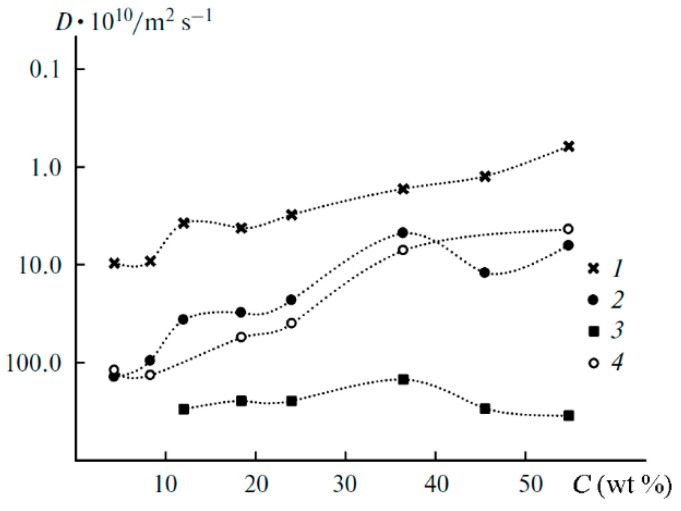
Dependences of the ^1^H self-diffusion coefficients (*D*) on the solvent concentration (*C*) for the fast (**1**), medium (**2**), and slow (**3**) components; (**4**) is the same for lithium cations [54].

As mentioned above, the diffusion decay of ^1^H for the samples containing more than 12 wt % EC is approximated by the sum of three exponential components described by Equation (2): fast (phase 1), medium (phase 2), and slow (phase 3) (Figure 20). The diffusion decays for the samples with a lower EC content show the biexponential shape. No spin echo signal is detected for the solvent-free polymers. The self-diffusion coefficient in phase 3 is almost independent of the solvent content since the samples contain 12 wt % EC (Figure 21, curve 3). The part of phase 3 is close to the ratio of the number of hydrogen atoms in the cyclic dimer of 2-hydroxyethyl acrylate to that in EC. Therefore, the slow component belongs to the molecules of 2-hydroxyethyl acrylate dimer, but the medium and the fast components (phases 1 and 2, respectively) are due to the solvent molecules. As shown in Figure 21, the self-diffusion coefficient of the medium component is close to that of lithium ions. Therefore, the medium component is due to the solvation of Li^+^ by EC molecules, while the fast one corresponds to the EC molecules connected to PEDA. These two types of molecules are exchanging, and the exchange time is about several hundreds of ms [54].

The results of the NMR study of PGE are consistent with the quantum-chemical modeling of PEDA-LiCLO_4_-EC complexes and the study by IR spectroscopy of the same PGE compositions (Table 1) [53,54].

The density functional theory studies of the energy and structures of mixed Li^+^ complexes and LiClO_4_ with EC and PEDA, modeled by oligomers H-((CH_2_)_2_COO(CH_2_)_2_O)_n_-CH_3_ (*n* ≤ 10), showed a stronger binding of the lithium-ion with the polymer matrix in the mixed complexes with one EC molecule at a low content of EC. This most likely resulted in a decrease in conductivity.

Less stable mixed complexes with three EC molecules can be formed with an increase in the EC fraction. They become unstable in EC excess because of the transition of the Li^+^ ions to solvate complexes containing only EC molecules [127]. A similar complex “Li^+^–4ECs and BF_4_^−^–4ECs solvate-separated ion pair” is shown in Figure 7a [121].

### 3.4. NMR in Gel Electrolytes with Ionic Liquid

Gel electrolytes with the ionic liquid addition are very perspective. The NMR investigations of these systems are given in [47,50,57,58,59,60,61,128].

Salt LiTFSI, IL 1-butyl-1-methylpyrrolidinium (Pyr_14_) TFSI and a variable amount of poly(ethylene oxide) electrolyte were studied by ^1^H, ^7^Li, ^19^F diffusion NMR and electrophoretic NMR (eNMR). It was shown that, depending on the composition, the mechanism of vehicular Li^+^ transport via anionic clusters shifts to the chain-dominated transport mechanisms. The lithium transport properties of the electrolytes based on the ionic liquid can be improved by the addition of PEO [50].

The 1-butyl-3-methylimidazolium tetrafluoroborate–propylene carbonate–ethylene carbonate, polyethylene glycol diacrylate polymer electrolyte, and LiBF_4_ salt system (PGE) were investigated by high resolution and pulsed-field gradient NMR.

The composition and conductivities are given in Table 6 [59].

**Figure 22:**
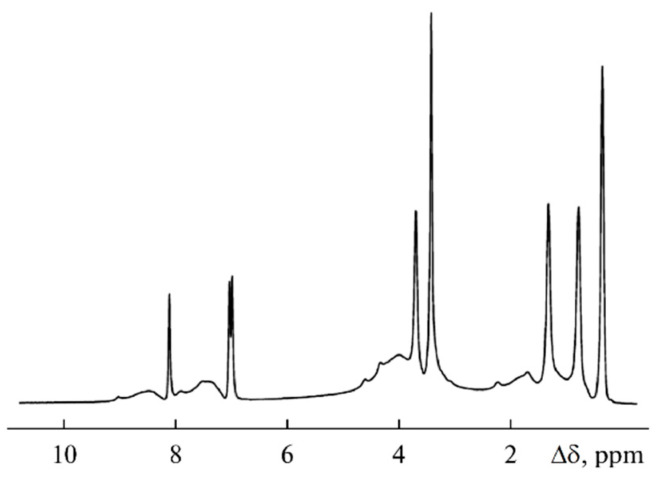
^1^H NMR spectra of sample 3 [58].

As shown in Figure 22, the ^1^H spectrum of compound 3 contains relatively narrow intense lines corresponding to the BMIBF_4_ ionic liquid and the broad signals of the PEG-DA polymer: -CH_2_-C(O)- 1.5–2.5 ppm; -CH_2_-O- 3.5–4.5 ppm; and -C=CH_2_ 7–9 ppm.

**Figure 23:**
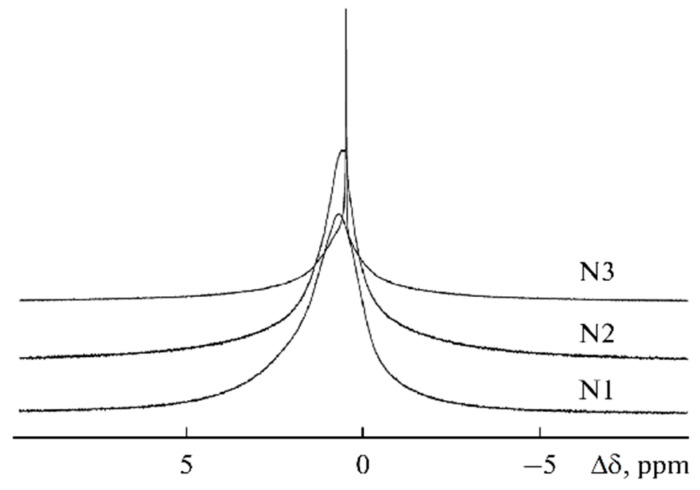
^7^Li NMR spectra of samples 1–3 [58].

The ^7^Li NMR spectra of samples 1–3 are presented in Figure 23. The linewidth decreases from sample 1 (270 Hz) to sample 3 (20 Hz), which indicates that the molecular mobility increases with increasing BMIBF_4_ content. The linewidth was 100, 30, and 50 for the propylene carbonate addition (samples 4, 5, and 6, respectively). The lines also narrowi with increasing ethylene carbonate addition from 50 to 15 Hz (7–9 samples).

The diffusion decays were approximated by one or two exponents. The biexponential shape indicates two phases with different self-diffusion coefficients (*D*_1_, *D*_2_) and populations (*p*_1_, *p*_2_) [59].

As shown in Table 6, only one type of Li^+^ coordination environment (solvation by the polymer matrix) is observed in the absence of organic solvent. As the 1-buthyl-3-methylimidazolium tetrafluoroborate content in the polymer increases from 44 to 64 wt % (compositions 2 and 3), the self-diffusion coefficients remain unchanged, but the conductivities increase by an order of magnitude. Two phases of lithium ions are formed with the addition of the solvent (compositions 4–9). The phase with the fast Li^+^ is likely due to the formation of complexes involving the solvent molecules. When propylene carbonate was added (from 15 to 31 wt % (compositions 4–6)), the conductivity also increased. The highest conductivity was achieved for composition no. 9, where the Li^+^ self-diffusion coefficients *D*_1_ and *D*_2_ are of the same order of magnitude: 4.1 × 10^−11^ and 1.2 × 10^−11^ m^2^/s. This fact indicates the substitution of ethylene carbonate molecules for the units of the polymer matrix in the Li^+^ ion coordination [53,127].

**Figure 24:**
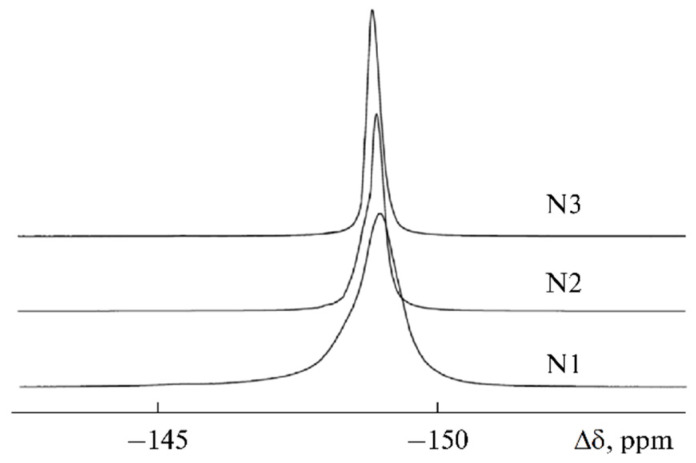
^19^F NMR spectra of samples 1–3 [58].

The ^19^F linewidth changed in a similar way (Figure 24). The line narrowed from 350 to 120 Hz for the samples from 1 to 3.

The self-diffusion coefficients of the anions containing ^19^F are presented in Table 4. As shown in Table 7, for composition 1 without solvent, only one low self-diffusion coefficient (6.1 × 10^−13^ m^2^/s) is observed, and the anions are likely immobilized on the polymer matrix. With an increase in the ionic liquid fraction, highly mobile anions appear with a self-diffusion coefficient of about 10^−11^ m^2^/s. With the addition of solvent compounds, the self-diffusion coefficient increased from 6.0 × 10^−12^ to 2.2 × 10^−11^ m^2^/s (propylene carbonate) and from 8.6 × 10^−12^ to 3.2 × 10^−11^ m^2^/s (ethylene carbonate). It should be noted that the self-diffusion coefficients of the anions are comparable to those of lithium cations (compositions 4–9) in spite of the fact that the anion size is larger than the cation size. This can be caused by cation solvation by solvent molecules.

The network polymer gel electrolyte matrix based on polyethylene glycol diacrylate in a medium of ionic liquid EMIBF_4_ and LiBF_4_ with PC and EC was investigated. The polymer compositions are given in Table 8 [60].

**Figure 25:**
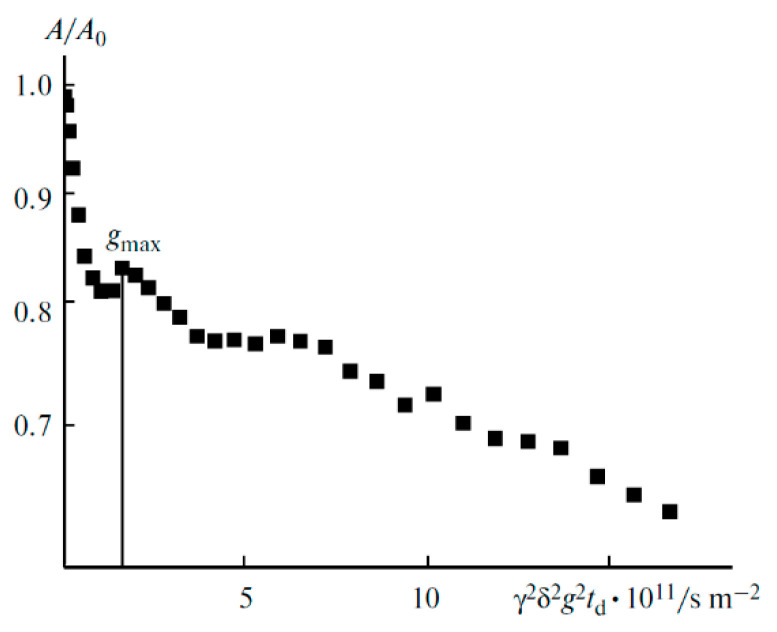
Diffusion decay on ^7^Li for the composition of PE 1 [60].

For polymer electrolytes without solvent, the diffusion decay is characterized by a periodic oscillation of the spin-echo amplitude (beats), which indicates a periodical spatial diffusion restriction (Figure 25). This restriction size is about 3.5 µm. The beats disappeared with increasing ionic liquid content and solvent additions, followed by increasing ^1^H and ^19^F self-diffusion coefficients and ionic conductivity by 2–3 orders of magnitude and by an order of magnitude for Li^+^ cations (Table 1 and Table 2).

**Figure 26:**
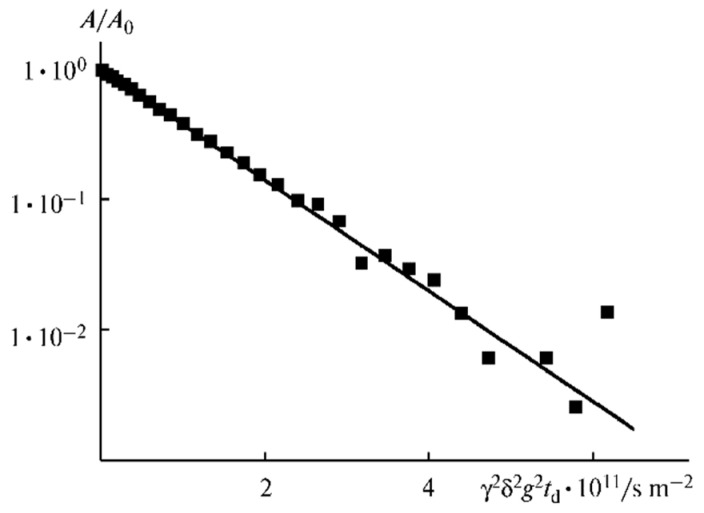
Diffusion decay on ^7^Li for the composition of PE 5 [60].

Thus, a periodical network is initially formed in the polymer. Ionic liquid insertion decreases the linkage density, and the ionic and molecular mobilities increase. Solvent addition causes further network loosening, and restriction disappears, followed by the exponential diffusion decay (Figure 26). Ionic liquid ions and BF_4_^−^ anions possess the highest mobility.

Self-diffusion coefficients of ^7^Li^+^ cations in the cross-linked polymer gel electrolytes based on PEG DA-EMIBF_4_-LiBF_4_ and the sample compositions are given in Table 8 [61]. As shown in Table 8, a small amount of solvent addition (EC and PC) results in phase (10% population) formation with high lithium cation self-diffusion coefficients *D_s_*_2_ (*D_s_*_2_ = 4.0 × 10^−10^ m^2^/s for sample 7 with EC and 2.5 × 10^−10^ m^2^/s for sample 4 with PC, respectively), which are two orders of magnitude higher than the self-diffusion coefficients *D*_s1_ of the main population phases. It may be assumed that the fast Li^+^ diffusion is due to the solvation of EC and PC complexes. At a higher solvent content, we observe the average self-diffusion coefficient as a result of a fast exchange between ^7^Li located in different environments, which increases with an increasing solvent amount (Table 8).

**Figure 27:**
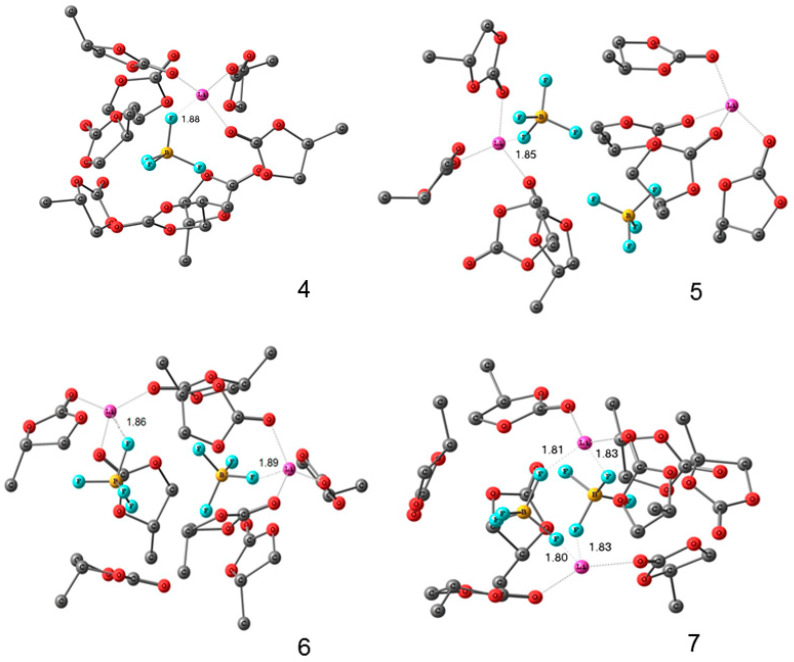
Calculated structures of the solvation complexes without ionic liquid components. Hydrogen atoms are omitted. Interatomic distances are given in Å [128].

**Figure 28:**
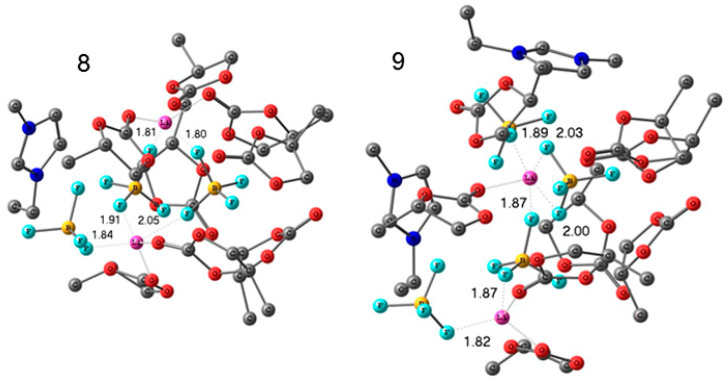
Calculated structures of the solvation complexes with the IL components. Hydrogen atoms are omitted. Interatomic distances are given in Å [128].

Lithium and 1-ethyl-3-methylimidazolium tetrafluoroborates (EMIBF_4_]) in PC solutions were studied by high-resolution ^1^H, ^7^Li, ^11^B, ^13^C, and ^19^F NMR. The degree of solvation of Li^+^ ions was also determined from a pulsed-field gradient ^1^H, ^7^Li, and ^19^F NMR measurements. The structures of Li^+^ solvation complexes with molecules of propylene carbonate and BF_4_^−^ anions and complexes associated with the ionic liquid were calculated using the density-functional theory. The calculated and experimental chemical shifts were compared. The structures of the complexes are shown in Figure 27 and Figure 28.

### 3.5. NMR in Nanocomposite Polymer Electrolytes

NMR studies in nanocomposite polymer electrolytes were considered in [49,65,72,73,77,92,101,104,105,106].

The mobility of the molecules containing ^7^Li and ^19^F was measured by pulsed-field gradient NMR and spin-lattice relaxation techniques in composite gel polymer electrolytes based on organo-modified montmorillonite clays. It was shown that the smectite clay surfaces solvate lithium and triflate ions, creating a preferential ion conduction pathway [49].

Solid-state ^7^Li NMR of a PCL-PTMC copolymer shows fast Li ions for the ^7^Li−^7^Li exchange between the phases of the polymer electrolyte LLZO compared to the PEO-based composite [133].

Two Li^+^-insulating oxide (fluorite Gd_0.1_Ce_0.9_O_1.95_ and perovskite La_0.8_Sr_0.2_Ga_0.8_-Mg_0.2_O_2.55_) polymer composite electrolytes were investigated by Li solid-state NMR. An increase in the Li+ ion (>10%) population results in a more mobile fraction in the composite electrolytes. This increase results from a strong interaction between the O_2_^−^ of the Li anions of the salt and oxygen of the surface oxide vacancies [73].

In poly(ethylene carbonate) and Li bis(fluorosulfonyl) imide, the influence of TiO_2_ nanoparticles on Li^+^ mobility was observed. The maximum values of Li^+^ self-diffusion and transport numbers were about 10^−11^ m^2^/s and 0.8, respectively, in the composites containing 1 wt % nanoparticles [101]. The highly conductive phase LiBH_4_, LiI with Al_2_O_3_ addition was investigated by ^1^H, ^6^Li, ^7^Li, ^11^B, and ^27^Al NMR [104]. An interaction of LiBH_4_-LiI with Al was shown by ^27^Al MAS NMR [105,106].

^7^Li NMR spectroscopy was applied to characterize the ion dynamics in quaternary composite solid-state electrolytes. The temperature dependences of the diffusion coefficients show two components consistent with the change in the morphology near the transition temperature where crystallinity was varied [77].

It was suggested based on ^1^H and ^7^Li NMR that the high ionic conductivity (0.5 mS/cm) and low activation energy (2.3 kJ/mol) of ion transport are due to grain boundaries between an excess of LiI and inert LiAlO_2_ ceramic nanoparticles in the composite polymer in the ceramic electrolytes [72].

The NMR study of the nanocomposite polymer electrolytes is discussed in detail below.

The effects of TiO_2_ (~60 nm) and Li_2_TiO_3_ (~20 nm) nanoparticles on the structure, conductivity, and self-diffusion in the polyether diacrylate–LiClO_4_–ethylene carbonate polymer gel electrolytes were studied. The compositions of the polymer electrolytes with nanoparticles additions are given in Table 9.

**Figure 29:**
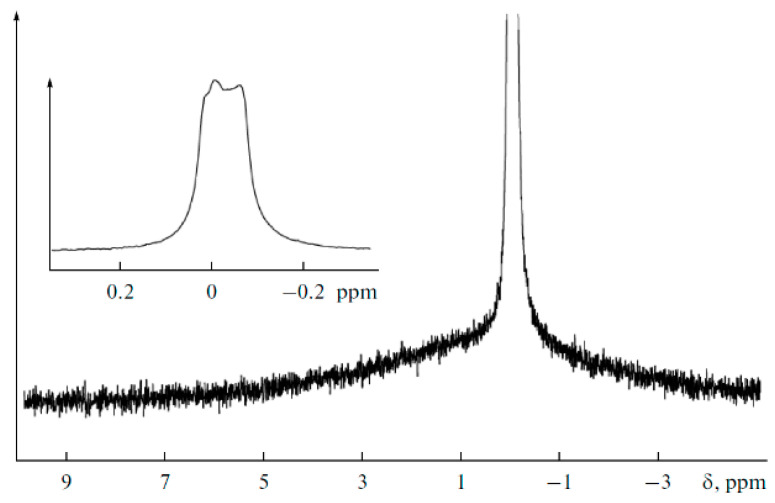
MAS ^7^Li NMR spectrum of the nanocomposite polymer electrolyte added with Li_2_TiO_3_. The sample spinning frequency is 10 kHz [92].

The ^7^Li NMR spectrum for the polymer electrolyte with Li_2_TiO_3_ (no. 3) is shown in Figure 29. The narrow line is a lithium perchlorate signal, while wide pedestals are due to lithium from nanoparticles. The narrow line consists of two lines attributed to different lithium positions (insert in Figure 29). The narrow line without splitting is observed for the polymer electrolyte with TiO_2_ addition.

**Figure 30:**
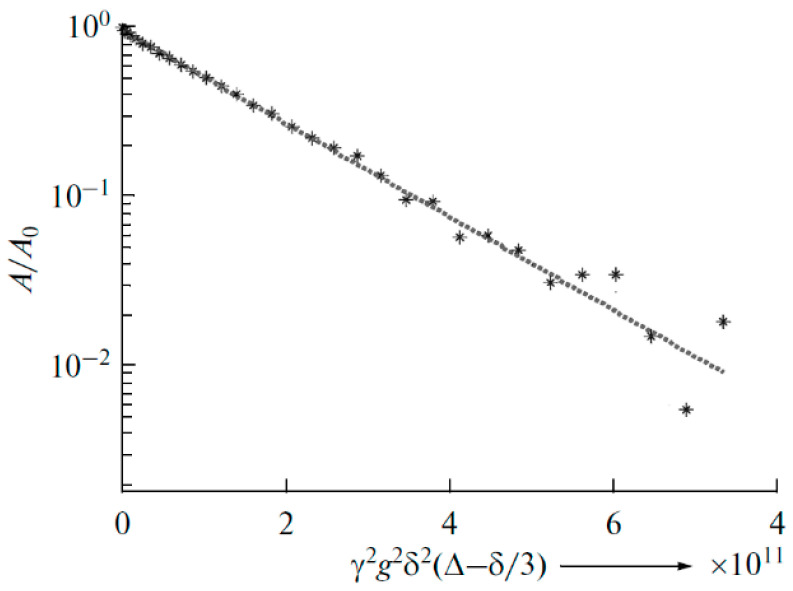
Diffusion decay of ^7^Li for polymer electrolyte no. 3 with Li_2_TiO_3_ nanoparticles (10 wt %) [92].

In polymer electrolyte no. 3 (Li_2_TiO_3_ addition), the exponential diffusion decay of ^7^Li is observed, which is characterized by only one self-diffusion coefficient (6.4 × 10^−12^ m^2^/s, Figure 30).

**Figure 31:**
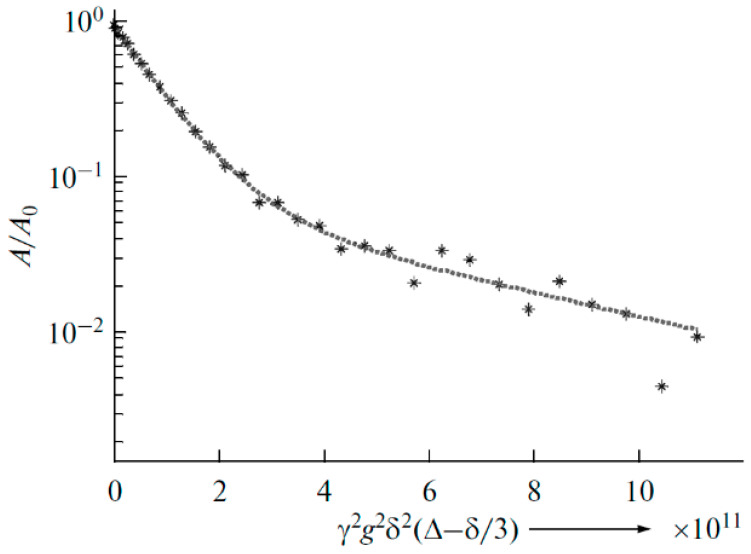
Diffusion decay of ^7^Li for polymer electrolyte no. 2 with TiO_2_ nanoparticles (10 wt %) [92].

For the polymer electrolyte with the addition of TiO_2_ nanoparticles, the biexponential diffusion decay of ^7^Li was recorded, which indicates two lithium surroundings or phases (Figure 31). The partial self-diffusion coefficients are 1.2 × 10^−11^ and 1.7 × 10^−12^ m^2^/s, and the phase populations are 0.9 and 0.1, respectively.

Some peculiarities of ion transport in the nanocomposite system based on a network matrix synthesized by radical polymerization of polyethylene glycol diacrylate in the presence of liquid aprotic electrolyte containing 1 M LiBF_4_ in γ-butyrolactone and SiO_2_ nanopowder were revealed. The compositions of the polymer electrolytes are given in Table 10.

**Figure 32:**
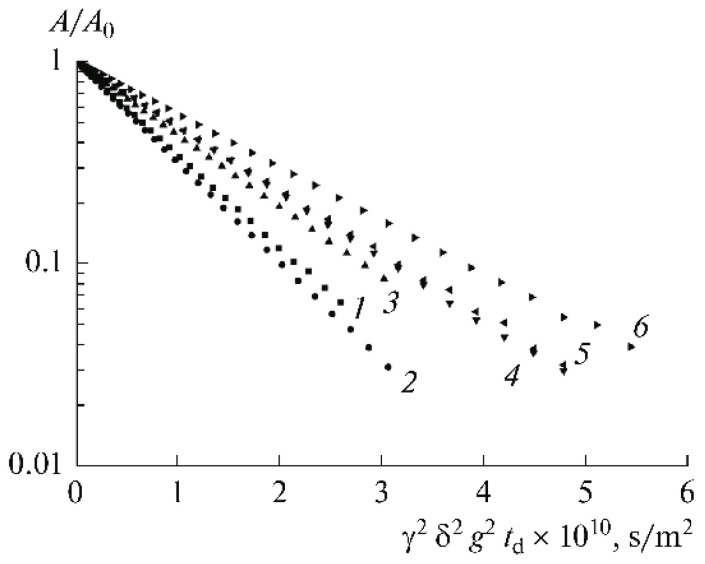
Dependence of amplitudes of spin-echo signals of ^7^Li on the squared amplitude of magnetic field gradient pulse (diffusion decay). The numbers correspond to the electrolyte composition numbers (Table 10) [65].

As shown in Figure 32, spin-echo ^7^Li attenuations (diffusion decays) are approximated by Equation (1). Therefore, lithium cation diffusion is characterized by only one self-diffusion coefficient.

Lithium-ion self-diffusion coefficients *D_s_* and ionic conductivities *σ* depend on the SiO_2_ content in the electrolyte (Table 10).

The highest self-diffusion coefficient (1.2 × 10^−10^ m^2^/s) corresponds to 2 wt % SiO_2_. This composition also shows the highest transport number of lithium cations (0.49). An increase in the number of mobile charge carriers is very likely due to salt dissociation. The second maximum of conductivity at 6 wt % SiO_2_ (no. 4) of the nanopowder content may be explained by the contribution of nanoparticle packing and the formation of additional pathways favorable for ion transport.

**Figure 33:**
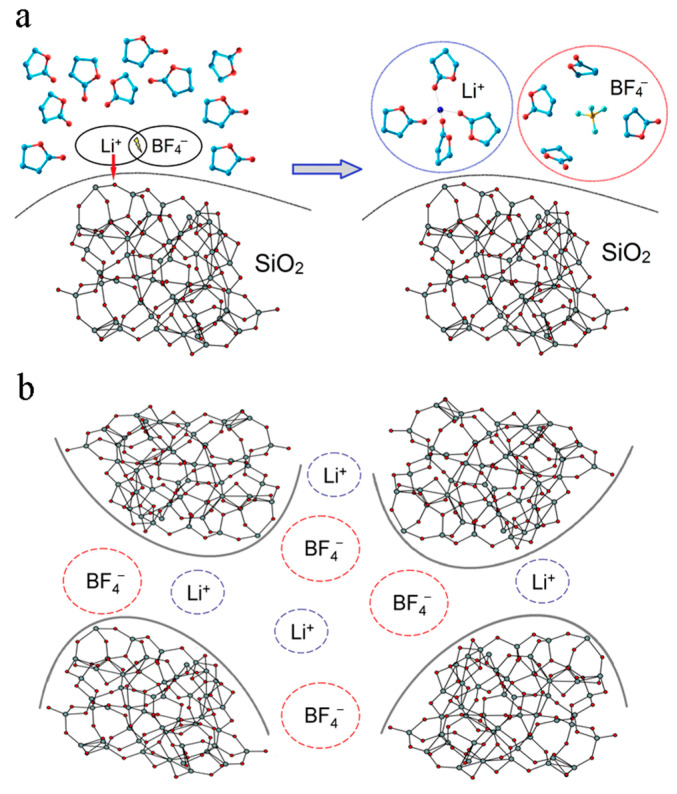
Scheme of ionic transport in the nanocomposite polymer electrolyte: (**a**) mechanism of LiBF_4_ salt dissociation to ions involving SiO_2_ surface groups; (**b**) ion transport over the surface of nanoparticles. Solvate shells of ions and ion pairs are designated by oblong dashed circles [65].

The scheme of ionic transport is given in Figure 33.

### 3.6. Nuclear Magnetic Resonance Study of Sodium-Ion Electrolytes

Batteries involving sodium salts are desirable because they are readily available at a low cost. On the one hand, ^23^Na NMR spectroscopy should be more sensitive to Na+ cation surroundings because the sodium nuclear chemical shift varies in some tenth ppm compared to ^7^Li chemical shift variations (1–2 ppm). On the other hand, the spin-spin relaxation time of ^23^Na is short and, therefore, it is difficult to measure the self-diffusion coefficient by pulsed-field gradient NMR. At the present time, only some papers are devoted to ^23^Na NMR in polymer electrolytes.

**Figure 34:**
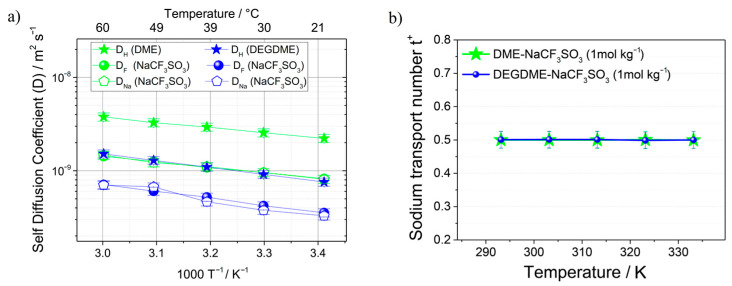
(**a**) Self-diffusion coefficients of ^1^H, ^19^F and ^23^Na in the DME-NaCF_3_SO_3_ (1 mol/kg) and DEGDME-NaCF_3_SO_3_ (1 mol/kg) electrolytes determined from 20 °C to 60 °C by PFG-NMR. (**b**) Sodium transport number (*t*_+_) calculated from the self-diffusion coefficients measured by PFG-NMR [135].

LiPF_6_ and NaPF_6_ salts solutions in glycol dimethoxy ethers (glymes) were studied [134,135]. The self-diffusion coefficients for ^7^Li, ^23^Na, and ^19^F in monoglyme (G1), diglyme (G2), and tetraglyme (G4) decrease from 10^−9^ m^2^/s in G1 to 10^−11^ m^2^/s in G4 [134]. The conductivities calculated from the Nernst–Einstein equation were compared with the measured values, and the degree of ion association was determined. The electrolytes show ion pairing with increasing temperature, which is explained by decreasing solvent dielectric constant with temperature. It was shown that ion association decreases if the solvent molecular size increases. The self-diffusion coefficients of ^1^H, ^19^F, and ^23^Na of NaCF_3_SO_3_ solutions in dimethoxyethane (DME) and diethylene glycol dimethyl ether (DEGDME) were measured by PFG NMR [135]. The temperature dependences of DH, DF, and DNa self-diffusion coefficients and sodium transport number (*t*_+_) calculated from the self-diffusion coefficients are shown in Figure 34. The DME and DEGDME electrolytes show suitable characteristics for sodium batteries, where the ion conductivity is about 10^−3^ S/cm, but the sodium transport number is 0.5. A DEGDME solution is more suitable for sodium battery applications

**Figure 35:**
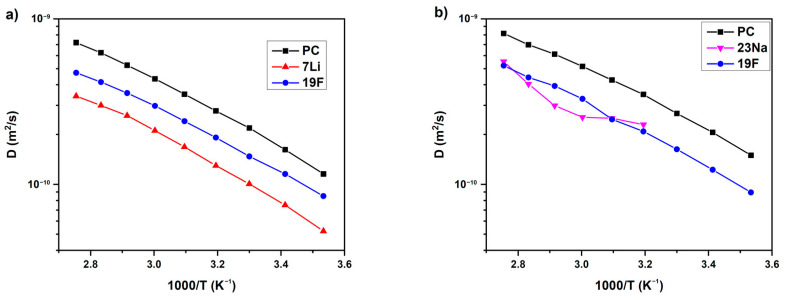
Temperature dependences of the NMR self-diffusion coefficients for the LiTFSI-0%G (**a**) and NaTFSI-0%G (**b**) electrolytes (without PAMAM). Solid lines are guides for the eye [136].

The PAMAM (Poly(amidoamine)) dendrimers dissolved in propylene carbonate (PC) together with LiTFSI or NaTFSI salts were studied by PFG ^7^Li, ^19^F and ^23^Na NMR [136]. The dependences of the self-diffusion coefficients on temperature and PAMAM content were analyzed. The temperature dependences of PC, ^7^Li, ^19^F and PC, ^23^Na, and ^19^F self-diffusion coefficients for LiTFSI and NaTFSI solutions without PAMAM are shown in Figure 35.

**Figure 36:**
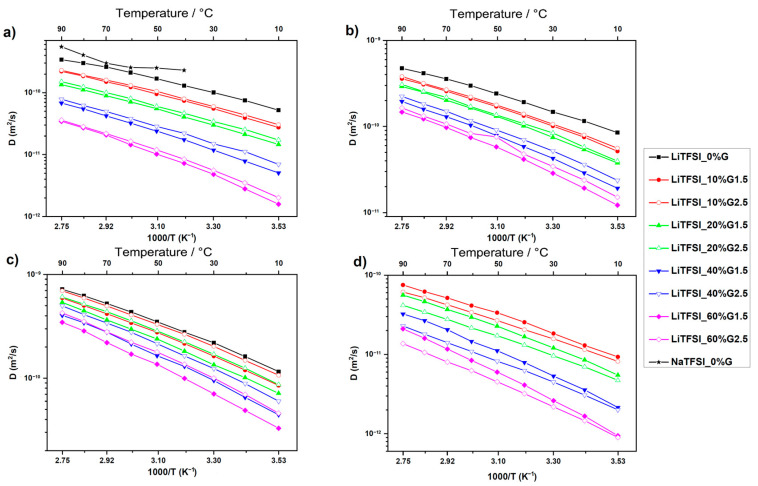
Temperature dependences of the NMR self-diffusion coefficients for the LiTFSI-based electrolytes for Li cation (**a**), TFSI anion (**b**), PC molecules (**c**), and PAMAM molecules (**d**). Solid lines are guides for the eye [136].

The effect of PAMAM on the diffusivity of Li^+^ cation (a), TFSI^-^ anion (b), PC molecules (c), and PAMAM molecules (d) in LiTFSI containing electrolytes is demonstrated in Figure 36.

The self-diffusion coefficients decrease by orders of magnitude with an increase in the PAMAM content due to increased viscosity. The self-diffusion coefficients of the solvent, cations, and anions are slightly higher in the samples with a high PAMAM generation, which is explained by weaker interactions of the PAMAM dendrimer with the cations, anions, and PC or a poor penetration of a solution into PAMAM G2.5 compared to PAMAM G1.5. A decrease in the Li cation self-diffusion coefficient compared with the TFSI^-^ anion and PC is observed. The diffusion of lithium cations is slower than that of a large anion. The TFSI lithium self-diffusion coefficient decreases with an increase in the concentration of PAMAM because of cation–dendrimer interactions.

## 4. Conclusions

The first significant half of the review (Section 2) is devoted to the compositions and conductivity of various types of electrolytes for lithium-ion batteries developed over the past decade. The compositions and conductivity of liquid electrolytes and solid, gel, and nanocomposite polymer electrolytes are considered in the second part of the review.

First, the compositions and conductivity of the liquid electrolytes are considered. The conduction mechanisms in the polymer gel electrolytes that contain them should be understood. Ion transport occurs through the liquid phase in gel systems.

Second, the compositions and conductivity of the solid polymer electrolytes are briefly considered. Gel electrolytes are discussed in the next section. They compose the most considered class of polymer electrolytes. They contain both aprotic solvents and ionic liquids. In the last paragraph, the nanocomposite electrolytes have found consideration. Here it is necessary to distinguish the composition of the polymer–salt–nanoparticle and polymer–salt–nanoparticle–solvent.

NMR applications for the polymer electrolytes are described in the third part.

High-resolution solid-state NMR, especially magic angle spinning (MAS) NMR, NMR relaxation, and pulsed-field gradient NMR applications for ion coordination and mobility, are discussed. Primary attention is given to gel electrolytes.

Section 3.1 is devoted to solid-state NMR, pulse field gradient NMR, exchange spectroscopy, cross-polarization, and rotational echo double-resonance applications to characterize the molecular structure and dynamics in solid polymer and hybrid electrolytes.

In Section 3.2, solutions of LiBF_4_ and LiClO_4_ in ethylene carbonate (EC) were studied as model systems by high-resolution ^1^H, ^7^Li, ^11^B, ^13^C, ^17^O, and ^35^Cl NMR spectroscopy. The dependence of the multinuclear chemical shifts on the solution concentration was analyzed. The quantum-chemical calculations of nuclear chemical shifts were performed. The structure models of cation-anion-solvent complexes depending on the concentration were proposed on the basis of the comparison of the experimental and calculated chemical shifts. Some examples of complex structures are shown in Figure 6 and Figure 7.

In Section 3.3, these electrolytes in the polymer network are discussed. In a polyester diacrylate (PEDA)–solvent system, two ^7^Li NMR lines were observed, and their chemical shifts and widths depended on the EC content. It was concluded that the narrow line was due to mobile Li^+^ coordinated by EC molecules, and the wide line belonged to Li^+^ that interacted with the polymer matrix. The ^7^Li NMR spectra of the composite electrolyte gel, poly(vinylidene fluoride-*co*-hexafluoropropylene) with the addition of silicate aerogel (SAG), ethylene carbonate, and propylene carbonate as solvents at different contents of aerogel and LiClO_4_, were analyzed. It may be concluded that Li^+^ cations interact with SAG, solvent, and PVDF-HPF. These interactions could favor the formation of transport conditions for the fast movement of Li^+^ cations over the SAG particles’ surface. The ion and solvent mobility in different spatial scales was investigated by NMR relaxation and pulsed-field gradient NMR techniques. It was assumed that the lithium cation macroscopic transfer is controlled by translational jumps over the distances compared with the sizes of the solvated cationic complexes. The comparison of the ionic conductivity calculated from the diffusion coefficient *σ*_NMR_ using the Nernst–Einstein equation with the experimental conductivity *σ*_sp_ enables the degree of dissociation *α* to be calculated as the ratio *α* = *σ*_sp_/*σ*_NMR_. The density functional theory studies of the energy and structures of mixed Li^+^ complexes and LiClO_4_ with EC and PEDA, which were modeled by oligomers H-((CH_2_)_2_COO(CH_2_)_2_O)_n_-CH_3_ (*n* ≤ 10), showed a stronger binding of the lithium ions with the polymer matrix in the mixed complexes with one EC molecule at a low content of EC resulting, most likely, in a decrease in the conductivity. Less stable mixed complexes with three EC molecules can be formed with an increase in the EC fraction. They become unstable in EC excess because of the transition of the Li^+^ ions to solvate complexes containing only EC molecules. A similar complex “Li^+^–4ECs and BF_4_^−^–4ECs solvate-separated ion pair” is shown in Figure 7a.

Gel electrolytes with the addition of ionic liquid and nanoparticles are very perspective. In Section 3.4, the 1-butyl-3-methylimidazolium tetrafluoroborate–propylene carbonate–ethylene carbonate, polyethylene glycol diacrylate polymer electrolyte and LiBF_4_ salt system (PGE) studied by high resolution and pulsed-field gradient NMR are described. Lithium and 1-ethyl-3-methylimidazolium tetrafluoroborates (EMIBF_4_]) in PC solutions were investigated by high-resolution ^1^H, ^7^Li, ^11^B,^13^C, and ^19^F NMR. The degree of solvation of Li^+^ ion was also determined from pulsed-field gradient ^1^H, ^7^Li, and ^19^F NMR measurements. Only one type of Li^+^ coordination environment (solvation by the polymer matrix) is observed in the absence of organic solvent. As the 1-buthyl-3-methylimidazolium tetrafluoroborate content in the polymer increases, the self-diffusion coefficients remain unchanged, but the conductivities increase by order of magnitude. Two phases of lithium ions are formed with the addition of the solvent. The phase with fast Li^+^ is likely due to the formation of complexes involving the solvent molecules. The anions containing ^19^F for the composition without solvent are characterized by a low self-diffusion coefficient (6.1 × 10^−13^ m^2^/s), and the anions are likely immobilized by the polymer matrix. When the fraction of the ionic liquid increases, highly mobile anions appear with self-diffusion coefficients of about 10^−11^ m^2^/s. It should be noted that the self-diffusion coefficients of the anions are comparable to those of lithium cations despite the anion size, which is larger than the cation size. This can be due to cation solvation by solvent molecules.

The structures of the Li^+^ solvation complexes with molecules of propylene carbonate and BF_4_^-^ anion and complex associated with the ionic liquid were calculated using the density-functional theory. The calculated and experimental chemical shifts were compared. The structures of the complexes are shown in Figure 27 and Figure 28.

In Section 3.5, the gel polymer electrolyte compositions with nanoparticles are considered. The effects of TiO_2_ (~60 nm) and Li_2_TiO_3_ (~20 nm) nanoparticles on the structure, conductivity, and self-diffusion in the polyether diacrylate–LiClO_4_–ethylene carbonate polymer gel electrolytes were studied. For the polymer electrolyte with TiO_2_ nanoparticles, the biexponential diffusion decay of ^7^Li was recorded, which indicates two lithium surroundings or phases. Ion transport particularities in the nanocomposite system based on a network matrix synthesized by radical polymerization of polyethylene glycol diacrylate in the presence of the liquid aprotic electrolyte containing 1 M LiBF_4_ in γ-butyrolactone and SiO_2_ nanopowder were revealed. The self-diffusion coefficients *D_s_* of lithium ions and ionic conductivities *σ* depend on the SiO_2_ content in the electrolyte. The highest self-diffusion coefficient (1.2 × 10^−10^ m^2^/s) corresponds to 2 wt % SiO_2_. This composition also shows the highest transport number of lithium cations (0.49). An increase in the number of mobile charge carriers is very likely due to salt dissociation. The second maximum of the conductivity at 6 wt % SiO_2_ of the nanopowder content can be explained by the contribution of nanoparticle packing and the formation of additional pathways favorable for ion transport. The scheme of ionic transport is given in Figure 33.

In Section 3.6, some results of the NMR study of sodium ion electrolytes are briefly presented. It was demonstrated that ^23^Na NMR is very informative for investigating these systems.

## Figures and Tables

**Table 1:** The compositions and conductivity of some solid polymer electrolytes.

№	Polymer	Salt	*σ*, S/cm(T_room_)	Ref.
1	PEO	LiPF_6_	10^−5^	[28]
2	Poly(ethylene oxide carbonate)	LiTFSI	7.4 × 10^−4^	[29]
3	Allil-PEO/Allil-Gallic acid	LiTFSI	4 × 10^−4^ at 60 °C	[30]
4	Polypropylene carbonate—PEO	LiTFSI	2 × 10^−5^	[31]
5	Crosslinked poly(tetrahydrofuran)	LiTFSI	1.2 × 10^−4^	[32]
6	PEG DA	LiClO_4_	3.5 × 10^−6^ at 60 °C	[24]
7	PEG MA	LiTFSI	2.13 × 10^−4^	[25]
8	Polyglycidyl MA-PEG	LiTFSI	2.1 × 10^−5^	[26]
9	PEG-DA-co-PEG MA	LiClO_4_	10^−5^	[27]
10	Polystyrene-b-PEO	LiTFSI	2.4 × 10^−4^	[33]
11	Cyclodextrin/PEO	LiTFSI	2.44 × 10^−6^	[34]

**Table 2:** Composition and characteristics of polymer gel electrolytes.

№	Polymer	Electrolyte Composition	*σ*, S/cm (*T_r_*_oom_)	*E*, V	Ref.
1	PVDF-HFP	LiBF_4_ in EC	1.7 × 10^−3^	-	[35]
	PVDF-HFP	LiBF_4_ in GBL	2.5 × 10^−3^	5.5	[36]
2	PVDF-HFP	LiPF_6_ in EC/DMC	3 × 10^−3^	-	[37]
3	c-PPO ^1^		0.8 × 10^−3^		
4	PBO ^2^		2 × 10^−3^		
5	PVDF-HFP	NaClO_4_ in EC/DMC/DEC	0.6 × 10^−3^	4.6	[38]
6	PVDF-HFP	LiPF_6_ in isobutyronitrile	17.2 × 10^−3^	-	[39]
7		LiTFSI in isobutyronitrile	10.8 × 10^−3^		
8		LiPF_6_ in PC	4 × 10^−3^		
9		LiTFSI in PC	5 × 10^−3^		
10	PVDF-HFP	LiPF_6_ in EC/DMC [Pyr_14_] ^3^ PF_6_	1.6 × 10^−3^	-	[40]
11	PE/PP/PVDF	LiPF_6_ in EC/DMC/EMC	3 × 10^−4^	4.8	[41]
12	PVDF	LiBF_4_ in PC	10^−4^	-	[42]
13	PVS ^4^/PVDF	LiTFSI in EC/PC	1.74 × 10^−3^	4.1	[43]
14	PVDF/PEO	LiClO_4_ in EC/PC	3.03 × 10^−3^	5.0	[44]
15	PVDF/methylcellulose/PVDF	LiPF_6_ in EC/DEM/EMC	1.5 × 10^−3^	-	[45]
16	PMMA	LiTFSI/LiBOB ^5^ in EC/PC	1.33 × 10^−3^	-	[46]
17	PMMA	Li[P4441] ^6^ [TFSI] Li[N4441] ^7^ [TFSI]	10^−4^	-	[47]
18	PECA/PET ^8^	LiPF_6_ in EC/DMC	2.54 × 10^−3^	4.7	[48]
19	PAN/PEO	LiTf in EC/PC	10^−3^	-	[49]
20	PEO	LiTFSI in Pyr_14_TFSI	10^−4^	-	[50]
21	PEO	LiTf in pentyl acetate	10^−5^	-	[51]
22	MATEMP ^9^	NaClO_4_ in EC/PC	5.13 × 10^−3^	5	[52]
23	PEDA	LiClO_4_ in EC	7.5 × 10^−4^	7	[53,54]
24	HBP ^10^	LiClO_4_ in PC	9 × 10^−4^	-	[55]
25	PAN elastomer	LiTFSI in PC	3.5 × 10^−3^	-	[56]
26	PEG-MA/BEMA ^11^	LiTFSI in Pyr_13_TFSI ^12^	2.5 × 10^−3^		[57]
27	PEG-DA	LiBF_4_ in EC/PC/BMIBF_4_^13^	2.5 × 10^−3^	-	[58,59,60,61,62,63,64,65,66,67,68,69,70,71,72,73,74,75,76,77,78,79,80,81,82,83,84,85,86,87,88,89]
30	PEG-DA	LiBF_4_ in EC/PC/EMIBF_4_^14^	4 × 10^−3^	-	[60,61]

^1^ c-PPO is poly [diemethyl-p-vinyl benzyl phosphonate-co-oligo (ethylene glycol) methacrylate] co-polymer. ^2^ PBO is poly [benzyl methacrylate-co-oligo (ethylene glycol) ether methacrylate)]. ^3^ Pyr_14_ is 1-butyl-1-methylpyrrolidinium. ^4^ PVS is polyvinylstyrene. ^5^ LiBOB is Lithium bisoxalato borate. ^6^ [P4441] is tributyl methyl phosphonium. ^7^ [N4441] is tributyl methyl ammonium. ^8^ PECA/PET is polyethyl-α-cyanoacrylate/poly (ethylene terephthalate). ^9^ MATEMP is di(2-methylacryloyltrioxyethyl) methyl phosphonate. ^10^ HBP is hyperbranched polymer based on methyl methacrylate and triethylene glycol dimethacrylate. ^11^ BEMA is bisphenol A ethoxylatedimethacrylate. ^12^ Pyr_13_ is N-Propyl-N-methylpyrrolidinium. ^13^ BMIBF_4_ is 1-butyl-3-methylimidazolium tetrafluoroborate. ^14^ EMIBF_4_ is 1-ethyl-3-methylimidazolium tetrafluoroborate.

**Table 3:** Compositions and properties of the nanocomposite polymer electrolytes.

№	Polymer	Electrolyte Composition	Nanoparticle	Conductivity, S/cm at *T*_298K_	Ref.
1	PEO	LiClO_4_	SiO_2_	1.1 × 10^−4^	[71]
2	PEO	LiI	LiAlO_2_	0.6 × 10^−3^	[72]
3	PEO	LiTFSI	Gd_0.1_Ce_0.9_O_1.95_	1.9 × 10^−4^	[73]
4	PEO	LiTFSI	perovskite La_0.8_Sr_0.2_Ga_0.8_Mg_0.2_O_2.55_	1.3 × 10^−4^	[73]
5	PEG-DA	LiTFSI	ZrCl_4_	8 × 10^−6^	[74]
6	PEG MA—PEG DA	LiClO_4_	SiO_2_	3.8 × 10^−5^	[75]
7	PVDF-HFP	LiTFSI	Li_0.33_La_0.557_TiO_3_/Li_3_PO_4_	5.1 × 10^−4^	[76]
8	PEO	LiTFSI, succinonitrile	Li_0.33_La_0.557_TiO_3_	10^−3^	[77]
9	PEO	LiClO_4_ in EC	SiO_2_	0.2 × 10^−3^	[78]
10	PEO/PEG	LiCF_3_SO_3_, dioxyphthalate	Al_2_O_3_	7.6 × 10^−4^	[79]
11	PEG_500_	LiCF_3_SO_3_, DME	LiNO_3_, TiO_2_	1 × 10^−3^	[80]
12	PVA ^1^/chitosan	LiClO_4_	TiO_2_	2.5 × 10^−3^	[81]
13	PVDF-HFP	LiPF_6_ in EC/DEC	SiO_2_	0.6 × 10^−3^	[82]
14	PVDF-HFP	LiBF_4_ in GBL	SiO_2_	3.7 × 10^−3^	[36]
15	PVDF-HFP	LiTFSI in EC/DMC	ZnS	3.3 × 10^−3^	[83]
16	PVDF-HFP	NaPF_6_ in EC/PC	TiO_2_	1.3 × 10^−3^	[84]
17	PVDF-HFP	NaTf in EC/PC	SiO_2_	4 × 10^−3^	[85]
18	PVDF-HFP	NaCF_3_SO_3_/BMICF_3_SO_3_^3^	TiO_2_	0.4 × 10^−3^	[86]
19	PVC/PEMA ^2^	LiClO_4_ in PC	TiO_2_	7.1 × 10^−3^	[87]
20	PPO-PEO-PPO ^4^	LiTFSI in EC/DMC	SiO_2_	1.32 × 10^−3^	[88]
21	P(MMA-*co*-BA)/PE ^5^	1M LiPF_6_ in EC/DMC	SiO_2_	2.26 × 10^−3^	[89]
22	PMMA	LiClO4 in PC	TiO_2_	3 × 10^−4^	[90]
23	PEDA	LiClO_4_ in EC	TiO_2_	1.8 × 10^−3^	[91,92]
24	PEDA	LiClO_4_ in EC	Li_2_TiO_3_	7 × 10^−4^	[92,93]
25	Polyester-polycarbonate	LiTFSI	ZrO_2_	~10^−3^	[93]
26	PEG-DA	LiBF_4_ in GBL	SiO_2_	4.3 × 10^−3^	[94]

^1^ PVA is polyvinyl alcohol. ^2^ PVC/PEMA is poly (vinyl chloride)/poly(ethyl methacrylate). ^3^ BMICF_3_SO_3_ is 1-butyl-3-methylimidazolium trifluoromethanesulfonate. ^4^ PPO is poly(propylene oxide). ^5^ P(MMA-*co*-BA)/PE is poly(methyl methacrylate-*co*-butylacrylate).

**Table 4:** Compositions of the polymer electrolytes.

PGE Composition No.	PGE Starting Components, wt %			
	PEDA	EC	LiClO_4_	Benzoyl Peroxide
1	90.5	-	8.1	1.4
2	86.6	4.3	7.8	1.3
3	82.9	8.3	7.5	1.3
4	79.7	12.0	7.1	1.2
5	73.8	18.4	6.7	1.1
6	68.7	24.0	6.3	1.0
7	54.6	36.4	7.6	1.4
8	45.6	45.5	7.5	1.4
9	36.5	54.8	7.3	1.4

**Table 5:** Self-diffusion coefficients of lithium *D*_Li_, calculated conductivity *σ*_NMR_, activation energies of self-diffusion *E*_a_, and conduction *E*_a_(*σ*_sp_) at different ethylene carbonate concentrations [53].

PGE Composition No.	[EC], wt %	[Li^+^]: [EC] in PGE	*D*_Li_, cm^2^/s (30 °C)	*σ*_NMR_, S/cm (30 °C)	*E*_a_(*σ*_sp_), kJ/mol		*E*_a_ of Self-Diffusion, kJ/mol
					20–50 °C	60–90 °C	50–80 °C
1	-	1:0			62.3 ± 1.7	48.7 ± 3.3	63.6
2	4.3	1.5:1	8.3 × 10^−9^	4.42 × 10^−5^	81.8 ± 2.2	57.7 ± 4.2	55.9
3	8.3	1:1.4	7.4 × 10^−9^	3.70 × 10^−5^	72.1 ± 0.4	50.7 ± 2.6	50.7
5	18.4	1:3.4	1.8 × 10^−8^	7.71 × 10^−5^	47.3 ± 3.0	33.0 ± 1.2	42.8
6	24.0	1:4.7	2.5 × 10^−8^	9.75 × 10^−5^	45.9 ± 1.4	28.3 ± 1.5	40.4
7	36.4	1:5.8	1.4 × 10^−7^	6.41 × 10^−4^	26.6 ± 2.1	12.0 ± 1.8	30.0
9	54.8	1:9.0	2.3 × 10^−7^	9.59 × 10^−4^	20.9 ± 0.9	9.9 ± 0.3	28.5

**Table 6:** Composition, conductivity (*σ*), and self-diffusion coefficients (*D*_s_) of PE measured by the PFG ^7^Li NMR technique.

No.	Ratio of PE Components, mol					*σ*/S·cm^−1^ (20 °C)	*D*_1_ m^2^/s	*p*_1_	*D*_2_, m^2^/s	*p*_2_
	PEG DA	LiBF_4_	BMIBF_4_	PC	EC					
1	1	1	1	–	–	2.9 × 10^−6^	-	-	-	-
2	1	1	2.5	–	–	2.4 × 10^−5^	4.5 × 10^−12^	1	-	-
3	1	1	6.5	–	–	1.4 × 10^−4^	4.8 × 10^−12^	1	-	-
4	1	1	2.5	2.2		2.4 × 10^−4^	4.5 × 10^−13^	0.76	2.2 × 10^−11^	0.24
5	1	1	2.5	3.5		6.9 × 10^−4^	1.7 × 10^−12^	0.89	5.7 × 10^−11^	0.11
6	1	1	2.5	5.6		8.5 × 10^−4^	2.1 × 10^−13^	0.93	6.1 × 10^−11^	0.07
7	1	1	5.6	–	3.4	8.1 × 10^−4^	2.6 × 10^−12^	0.77	2.4 × 10^−11^	0.23
8	1	1	4.5	–	5.6	1.2 × 10^−3^	4.2 × 10^−12^	0.78	2.6 × 10^−11^	0.22
9	1	1	6.0	–	9.5	2.5 × 10^−3^	1.2 × 10^−11^	0.83	4.1 × 10^−11^	0.17

**Table 7:** ^19^F self-diffusion coefficients [59].

No.	*D*_1_, m^2^/s	*p*_1_	*D*_2_, m^2^/s	*p*_2_
1	6.07 × 10^−13^	1	–	–
2	7.91 × 10^−13^	0.7	1.73 × 10^−11^	0.33
3	2.60 × 10^−12^	0.63	1.27 × 10^−11^	0.39
4	6.00 × 10^−12^	1	–	–
5	1.79 × 10^−11^	1	–	–
6	2.2 × 10^−11^	1	–	–
7	8.57 × 10^−12^	1	–	–
8	1.71 × 10^−11^	1	–	–
9	3.24 × 10^−11^	1	–	–

**Table 8:** Compositions, conductivity (*σ*), and self-diffusion coefficients (*D*_s_) of PE measured by the PFG ^1^H, ^7^Li, and ^19^F NMR technique (*T* = 24 °C). Ratio PEG DA/LiBF_4_ =1/1 mol [60,61].

No.	Ratio of PE Components, mol			*σ*/S·cm^−1^ (20 °C)	*D*_s_/m^2^·s^−1^		
	EMIBF_4_	EC	PC		^1^H	^7^Li *	^19^F
1	1	–	–	1.4 × 10^−6^	5.8 × 10^−13^	2.0 × 10^−13^	3.6 × 10^−13^
2	2.5	–	–	7.4 × 10^−5^	2.5 × 10^−12^	5.5 × 10^−13^	1.5 × 10^−12^
3	6.5	–	–	2.6 × 10^−3^	2.5 × 10^−11^	5.0 × 10^−12^	1.8 × 10^−11^
4	6	–	3	2.6 × 10^−3^	3.0 × 10^−11^	5.2 × 10^−12^ (0.9) 2.5 × 10^−10^ (0.1)	2.3 × 10^−11^
5	6	–	6	3.6 × 10^−3^	4.0 × 10^−11^	9.6 × 10^−12^	3.6 × 10^−11^
6	6	–	9	4.4 × 10^−3^	4.4 × 10^−11^	1.3 × 10^−11^	4.1 × 10^−11^
7	6	3	–	2.1 × 10^−3^	3.0 × 10^−11^	6.1 × 10^−12^ (0.9), 4.0 × 10^−10^ (0.1)	2.5 × 10^−11^
8	6	6	–	3.9 × 10^−3^	3.2 × 10^−11^	1.3 × 10^−11^	4.2 × 10^−11^
9	6	9	–	4.2 × 10^−3^	5.1 × 10^−11^	1.3 × 10^−11^	4.8 × 10^−11^

* Phase population with a given *D*_s_ is indicated in parentheses.

**Table 9:** Compositions of the polymer electrolytes added with nanoparticles [92].

No. of Polymer Electrolyte	Polymer Electrolyte Initial Components, wt %			
	PEDA:EC (1:1)	LiClO_4_	Nanodisperse Filler	Benzoyl Peroxide
1	91.1	7.5	–	1.4
2	81.1	7.5	TiO_2_—10.0	1.4
3	81.1	7.5	Li_2_TiO_3_—10.0	1.4

**Table 10:** Parameters of ion transport for different NPE compositions: conductivity (*σ*_sp_) at 20 °C, effective activation energies of conduction (*E*^ef^_a_ (*σ*_sp_)), self-diffusion coefficients (*D*_s_), and transport numbers with respect to lithium cations (*t*_+_) [65].

No.	Starting Components of NPE, wt %				*t*_+_	*D*_s_, 10^−10^ m^2^/s (24 °C)	*σ*, mS/cm (20 °C)
	PEG-DA_575_	1 M LiBF_4_ in GBL	SiO_2_	BP			
1	15	84	0	1	0.44	1.10	3.24
2	15	82	2	1	0.49	1.20	3.76
3	15	80	4	1	0.34	0.83	3.35
4	15	78	6	1	0.32	0.76	4.52
5	15	76	8	1	0.29	0.73	2.61
6	15	74	10	1	–	0.61	2.34

## Abbreviation

NaTf	sodium trifluoromethanesulfonate
NaTFSI	sodium bis(trifluoromethanesulfonyl) imide NaN(SO_2_CF_3_)_2_
LiTFSI	lithium bis(trifluoromethanesulfonyl) imideLiN(SO_2_CF_3_)_2_
LiTf	Lithiumtrifluoromethanesulfonate—LiSO_3_CF_3_
LiBOB	Lithium bisoxalato borate
BMICF_3_SO_3_	1-butyl-3-methylimidazolium trifluoromethanesulfonate
BMIBF_4_	1-butyl-3-methylimidazolium tetrafluoroborate
EMIBF_4_	1-ethyl-3-methylimidazolium tetrafluoroborate
[N4441] [TFSI]	tributyl methyl ammonium bis(trifluoromethanesulfonyl)imide
[P4441] [TFSI]	tributyl methyl phosphoniumbis(trifluoromethanesulfonyl)imide
[Pyr_14_]PF_6_	1-butyl-1-methyl-pyrrolidinium hexafluorophosphate
Pyr_13_TFSI	N-Propyl-N-methylpyrrolidinium bis(trifluoromethanesulfonyl)imide
Pyr_14_TFSI	N-butyl-N-methyl-pyrrolidinium bis(trifluoromethanesulfonyl) imide
BEMA	bisphenol A ethoxylatedimethacrylate
CD	cyclodextrin
DEC	diethyl carbonate
DMC	dimethyl carbonate
DME	1,2-dimethyl ether
EC	ethylene carbonate
EMC	ethyl methyl carbonate
GBL	gamma-butyrolactone
HBP	hyperbranched polymer based on methyl methacrylate and triethylene glycol dimethacrylate
IL	ionic liquid
IBN	isobutyronitrile
LE	liquid electrolyte
LIB	lithium ion battery
MATEMP	di(2-methylacryloyltrioxyethyl) methyl phosphonate
MTBE	*tert*-butyl methyl ether
NMR	Nuclear magnetic resonance
NPE	Nanocomposite polymer electrolyte
PFG NMR	Pulsed field gradient NMR
PAN	polyacrylonitrile
PAMAM	poly(amidoamine)
PBO	poly [benzyl methacrylate-co-oligo (ethylene glycol) ether methacrylate)]
PC	propylene carbonate
PE	polyethylene
PEDA	polyether diacrylate
PEG	polyethylene glycol
PEG-DA	poly(ethylene glycol) diacrylate
PEG-MA	poly(ethylene glycol) methacrylate
PEG-MEM	poly(ethylene glycol) methyl ether methacrylate
PEMA	poly(ethyl methacrylate)
PEO	polyethylene oxide
xPTHF	poly(tetrahydrofuran)
PGE	polymer gel electrolyte
PMMA	poly(methyl methacrylate)
P(MMA-*co*-BA)	poly(methyl methacrylate-*co*-butylacrylate)
PP	polypropylene
c-PPO	poly [diemethyl-p-vinyl benzyl phosphonate-co-oligo (ethylene glycol) methacrylate] co-polymer
PPO	poly (propylene oxide)
PVC	poly (vinyl chloride)
PVA	polyvinyl alcohol
PVDF	poly(vinylidene fluoride)
PVDF-HFP	poly(vinylidene fluoride-*co*-hexafluoropropylene)
PVS	polyvinylstyrene
SAG	silicate aerogel
SDC	Self-diffusion coefficients
SPE	Solid polymer electrolyte
THF	tetrahydrofuran
TMAN	trimethyl acetonitrile
